# Bimodal fluorogenic sensing of matrix proteolytic signatures in lung cancer†
†Electronic supplementary information (ESI) available. See DOI: 10.1039/c8ob01790e


**DOI:** 10.1039/c8ob01790e

**Published:** 2018-08-23

**Authors:** Alicia Megia-Fernandez, Bethany Mills, Chesney Michels, Sunay V. Chankeshwara, Nikola Krstajić, Chris Haslett, Kevin Dhaliwal, Mark Bradley

**Affiliations:** a School of Chemistry and the EPSRC IRC Proteus , University of Edinburgh , Joseph Black Building , David Brewster Road , Edinburgh , EH9 3FJ , UK . Email: Mark.Bradley@ed.ac.uk; b EPSRC Proteus Hub , MRC Centre of Inflammation Research , Queen's Medical Research Institute , University of Edinburgh , 47 Little France Crescent , EH16 4TJ , Edinburgh , UK . Email: Kev.Dhaliwal@ed.ac.uk; c Institute for Integrated Micro and Nano Systems , School of Engineering , University of Edinburgh , EH9 3JL , UK

## Abstract

Optical biosensing based on the activation of fluorescent reporters offers a powerful methodology for the real-time molecular interrogation of pathology. Here we report a first-in-class, bimodal fluorescent reporter strategy for the simultaneous and highly specific detection of two independent proteases (thrombin and matrix metalloproteases (MMPs)) pivotal in the fibroproliferative process surrounding lung cancer, based on a dual, multiplexing, peptide FRET system. This sophisticated synthetic smartprobe, with a molecular weight of 6 kDa, contains two independent fluorophores and quenchers that generate photonic signatures at two specific wavelengths upon activation by target enzymes within human lung cancer tissue.

## Abstract 2

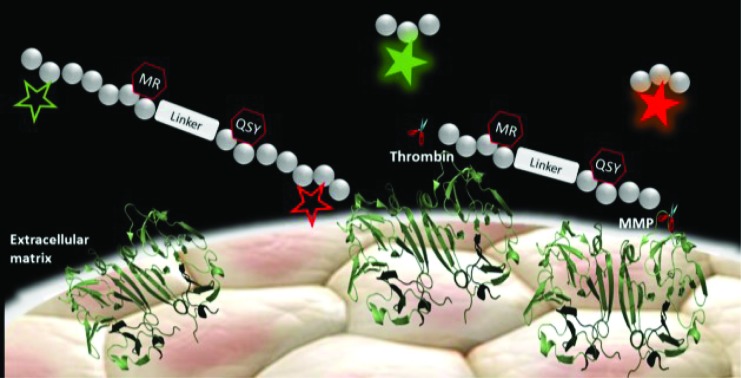
We present a highly-specific, rapidly activatable dual-FRET-smartprobe for the independent optical detection of metalloproteinases and thrombin in lung cancer tissue.

## Introduction

Lung cancer is the leading cause of cancer death in the world and most patients present with late stage disease due to a protracted diagnostic pathway.^1^ There have been major advances in interventional approaches for sampling suspected lung cancer in the distal lung that are augmented by routinely employed navigational technologies and the emergence of fibre based optical approaches.^2^–^4^ We have previously described the use of such platforms for guiding biopsy but a major limitation remains the lack of molecular probes to highlight suspected tumours and to guide sampling.^5^ Lung cancer is characterised by significant enzymatic activity in the penumbra and we sought to develop and validate a unique chemical probe with bimodal photonic amplification which had significant advantages over classical individual reporters.

Biosensing of enzymatic activity has been at the focus of significant chemical and clinical advances,^6^–^8^ with fluorescent probes reporting protease activity *in vivo*, including the cathepsins,^9^–^11^ caspases,^12^ enzymes of the coagulation cascade^13^ and those responsible for matrix remodelling.^14^ Many of these probes are peptide-based, often exploiting the FRET (Förster Resonance Energy Transfer) phenomenon to detect enzymatic activity.^13^,^15^ Optical-imaging probes based on fluorescence offer the possibility of optical multiplexing but previous reports utilizing fluorescent probes for multi-analyte detection have principally focused on the sequential activation of a single fluorophore with enzymes working in series^16^ or for the detection of metal ions and small molecules such as H_2_O_2_, NO or H_2_S,^17^,^18^ with the few reported examples of multienzymatic detection strategies relying on the turn-on of a single caged fluorophore.^19^

Our aim in this work was to develop the chemical tractability and advance a strategy for the simultaneous analysis of multiple molecular targets in lung cancer tissue which has significant advantages over independent sensors or sequential sensing. To date, there are no reports of probes that can independently report on two different enzyme activities simultaneously using a single chemical entity and at two different wavelengths, and no such multi-enzyme sensing has been reported in human lung cancer. To achieve this, we combined four optical entities (two fluorophores and two quenchers) in a single molecule to yield a biologically robust, highly sensitive and specific FRET dual-enzyme reporter, with the key deliverable of high substrate-specificity over by-stander enzymes, while functioning within the environment of *ex vivo* human diseased tissue.

The focus for our dual-enzyme sensor were key enzymes in the inflammatory cascade surrounding lung cancer,^20^–^22^ thrombin and matrix metalloproteinases-2, -9 and -13 (MMP-2, -9 and -13).^23^–^26^ Thrombin and the MMPs are two distinct classes of proteolytic extracellular proteins that play important temporal roles in fibroproliferation and coagulation,^20^–^22^ which may lead to dysregulation of the extracellular matrix (ECM). They have key roles in early carcinogenesis, in promoting early tumour–platelet interactions and have an association with pulmonary metastases. They are highly overexpressed in the malignant matrix and in fibrosis,^26^–^28^ and as such are rational biomarkers for the molecular imaging of the matrix response surrounding cancer.

## Experimental

### Ethics statement

All experiments using human samples *in vitro* were performed following approval of the appropriate regional ethics committee (REC), NHS Lothian (references 13/ES/0126 and 15/HV/013), and with informed consent of the patients. All murine experiments were performed under UK Home Office license 60/4434.

### Statistical analysis

Statistical analyses were performed using Prism 7 (GraphPad Software Inc., La Jolla, CA, USA). Where appropriate, analyses were performed using the student's *t*-test or one-way ANOVA. Unless otherwise stated error bars show standard error of the mean (s.e.m).

### Synthesis and characterization

Synthetic procedures and full characterization of compounds **1–5** and intermediates are described in the ESI.†

### Enzymatic validation of dual-probe **3**

To determine the enzymatic specificity of the dual-probes **1–5** (fragments **b** and **f**, and peptides undergoing optimisation as outlined in the ESI†) the probes were incubated at a concentration of 5 μM (unless otherwise stated) with the required recombinant MMP enzymes (Enzo Life Sciences) (active domains of MMP-1, -2, -3, -7, -8, -9, -10, -11, -12 and -13, 30 nM), thrombin (Sigma Aldrich) (5 U ml^–1^), Factor Xa (Sigma Aldrich) (500 nM), Plasmin (Sigma Aldrich) (30 nM), and human neutrophil elastase (30 nM). Where enzymes were added sequentially to dual-probe **3**, the first enzyme was incubated with the probe for 60 min prior to the addition of the second enzyme. Data was collected from 10 min incubation of probe with enzyme. Where appropriate enzymes were incubated with specific inhibitors for 30 min at 37 °C prior to addition of the dual-probe **3**. The pan-MMP inhibitor Marimastat (Tocris Biosciences) was used at 20 μM, while the thrombin inhibitor (anti-thrombin III (AT3, Sigma Aldrich)) was pre-incubated at 1 μM. Enzyme free reactions served as a control. Enzymatic reactions were performed in MMP buffer (10 mM CaCl_2_, 6.1 g Tris-HCl, 8.6 g NaCl per litre, pH 7.5) in a final volume of 20 μL. Reactions were performed in duplicate in 384 well plates (Life Technologies) with optically clear plate seals (Thermo Scientific) and repeated thrice (independently). Fluorescence signal was measured for up to 60 min, ex/em 485/528 nm (FAM) and ex/em 640/670 nm (Cy5) using microplate reader (BioTek Synergy H1 multi-mode reader). Data was normalised to buffer alone and are presented as fold-change in signal (Relative Fluorescent Units) compared to enzyme-free controls at 10 min. Data was plotted using Prism 7 (GraphPad Software Inc., La Jolla, CA, USA).

### Neutrophils and platelet rich plasma extraction

Platelet rich plasma (PRP) and neutrophils were isolated from the blood of healthy human volunteers as previously described.^29^ PRP was collected after initial centrifugation step of whole blood and stored at –20 °C until use. The number of retrieved neutrophils was determined with NucleoCounter NC-1000 (Chemo Metec). Neutrophils were resuspended at a concentration of 20 × 10^6^ mL^–1^ in 0.9% NaCl with 0.9 mM CaCl_2_. Cells were incubated for 30 min at 37 °C. Cells to be stimulated were subsequently activated with 5 μM calcium ionophore A23187 (Tocris Bioscience) for 30 min at 37 °C. Neutrophils were harvested by centrifugation at 400*g* for 5 min. Supernatants were removed and stored. Plate reader assays were performed as described above, with the MMP buffer replaced with neutrophil supernatant or PRP. All experiments were carried-out in duplicate using three independent donors (one donor per independent repeat). Data was normalised by background subtraction of intrinsic fluorescence.

### Atherosclerotic plaque imaging

Human carotid plaques were stored in 0.9% NaCl on ice immediately following excision. All samples were used within 2 h of retrieval. Carotid plaques were sliced in half lengthwise and placed into wells of a 6-well plate. 2 mL of 5 μM ice-cold dual-probe **3** was added to the well containing the carotid plaque, and an adjacent control well. Fluorescence intensity was imaged over 60 minutes using a Photon-Imager Optima System (Biospace Lab) set to detect FAM and Cy5 fluorescence. After 60 min, the plaque was removed from the well and the plate was imaged again to compare the fluorescent signal from within the well for sample and control wells. Images were analysed using M3 Vision software (Biospace Lab). Regions of interest (ROIs) were drawn around entire wells and fluorescence intensity p s^–1^ cm^–2^ sr^–1^ were determined. Data was collected from 3 independent carotid plaques. Solutions from the carotid plaque well and control wells were retrieved and analysed by MALDI-TOF MS.

### Lung tissue imaging

Human lung tissue (adenocarcinoma tumour and normal tissue from a site separate to the tumour location) were obtained following surgical resection and stored at –80° C until use. The tissue was sliced into 1 × 4 mm^2^ sections and placed in the wells of a 96-well plate with 100 μL MMP buffer. Where appropriate, marimastat (50 μM) and AT3 (1 μM) were added to the tissue and incubated at 37 °C for 60 min prior to the addition of dual-probe **3** (5 μM). The tissue was imaged over 30 min using a wide-field microendoscopy fluorescent imaging system as previously reported^30^ with a clinically approved commercial imaging fibre (Alveoflex, Mauna Kea Technologies). At each time point 10 s of images were taken with a 25 ms exposure time, at 10 frames per second, with LEDs aligned for FAM and Cy5 detection. Average fluorescence for each sample was calculated by determining the average fluorescence per frame for each time point (55 median frames out of 75 frames collected per time point). All images were brightness and contrast enhanced with the same parameters. A single patient specimen was collected and analysed.

### Murine LPS model

The murine LPS model is outlined in the ESI.†

## Results and discussion

### Design, synthesis and initial validation of the dual-probe

**Fig. 1:**
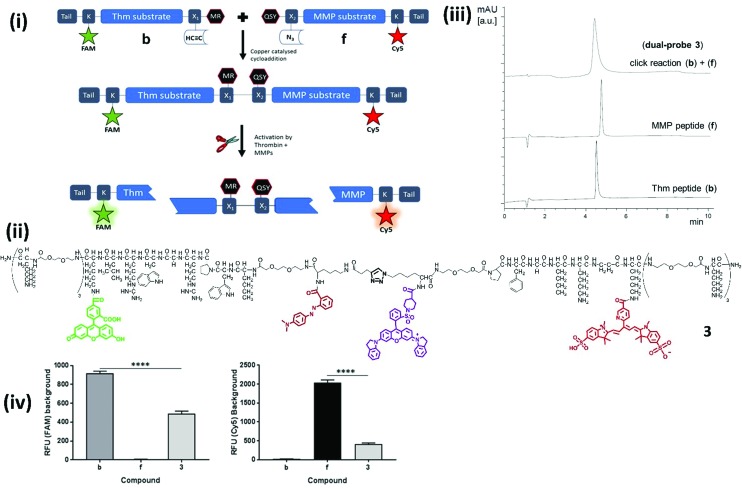
Design, synthesis, structures and quenching of the dual-probes. (i) Synthesis and mode of action of the dual-probe for thrombin (Thm, Table 1 – sequence 1) and matrix metalloproteases (MMP-2, -9 and -13, Table 1 – sequence 2). (ii) Full structure of compound **3**. For clarity the thrombin substrate (with associated X_1_ group and tail) is drawn in an unconventional C → N orientation. (iii) Reverse phase HPLC analysis (*λ* 500 nm) of the fragments (**b** and **f**) showing the rapid and clean conjugation to generate dual-probe **3**. (iv) The background fluorescence of fragments **b**, **f**, and dual-probe **3** were measured to determine the extent of FRET quenching for each of the fluorophores [ex/em 485/528 nm (FAM) and 640/670 nm (Cy5)]. Data is the mean of three independent replicates performed in duplicate. Error bars represent s.e.m. Statistical analysis was performed with a one-way ANOVA test, **** *P* < 0.0001.

Our novel FRET based dual-probe incorporated highly optimised thrombin and MMP peptide based-substrates (see ESI†), as well as two strategically placed fluorophores and two quenchers (Fig. 1), allowing the release of specific fluorescent fragments that could be detected at different wavelengths, with 5-FAM/Methyl Red and Cy5/QSY21 fluorophore/quencher pairs chosen for the thrombin and MMP fragments respectively. In addition, both fluorophores were strategically placed to ensure that following cleavage they remained within the liberated hydrophilic tail. The rationale for the chosen dye/quencher pairs were based upon their well differentiated emission wavelengths and minimal spectral overlap. It was considered for its use within the multi-colour microendoscopy imaging system (with appropriate LEDs for FAM and Cy5 excitation). A number of peptide sequences and scaffold structures were iterated and evaluated (Table 1) to yield the final optimised dual-probe **3** (Fig. 1ii). The peptide sequence for thrombin was selected following a lead optimisation programme;^31^ while the optimised peptide sequence for MMP-2, -9 and -13 was selected after extensive screening as detailed in the ESI.† The initial dual-probe synthesized (dual-probe **1**) was cleaved by thrombin and MMP-2, -9 and -13 but was shown to have very poor plasmin stability (Fig. S1†) a major issue, as this is an abundant protease *in vivo* and this protease undoubtedly cleaves many previously reported MMP probes. The MMP peptide sequence was developed to give dual-probe **2** (changing the peptide sequence from GPKGLKG to PFGNleKβA – see ESI†) which resulted in plasmin stability whilst preserving target MMP cleavage specificity. However non-specific cleavage of the thrombin peptide in dual-probe **2** by plasmin was still observed (Fig. S1†), thus this dual-probe underwent further iteration, substituting the l-leucine residue with d-leucine (NleWPRGWR(D)L) to yield the plasmin inert but thrombin responsive, dual-probe **3**.

**Table 1:** Showing the peptide sequences and spacers incorporated into dual-probe scaffold

Compound	Sequence **1** (thrombin substrates)	Sequence **2** (MMP substrates)	X_2_
**1**	–NleWPRGWRL	–GPKGLKG–	–K(PEG_2_)-PEG_2_–
**2**	–NleWPRGWRL–	–PFGNleKβA–	–K(PEG_2_)-PEG_2_–
**3** ^*a*^	–NleWPRGWR(d)L–	–PFGNleKβA–	–K-PEG_2_–
**4** ^*a*^	–NleWP(d)RGWR(d)L–	–PFGNleKβA–	–K-PEG_2_–
**5** ^*a*^	–NleWPRGWR(d)L–	–PFG(d)NleKβA–	–K-PEG_2_–

^*a*^ d-Aminoacids d-Leu, d-Arg and d-Nle were used where indicated. **X**_**1**_: –PEG_2_-K(–CO(CH_2_)_2_–C≡CH). **Tail:** -[PEG_2_-(d)Lys]_3_-NH_2_; Nle:Norleucine.

**Fig. 2:**
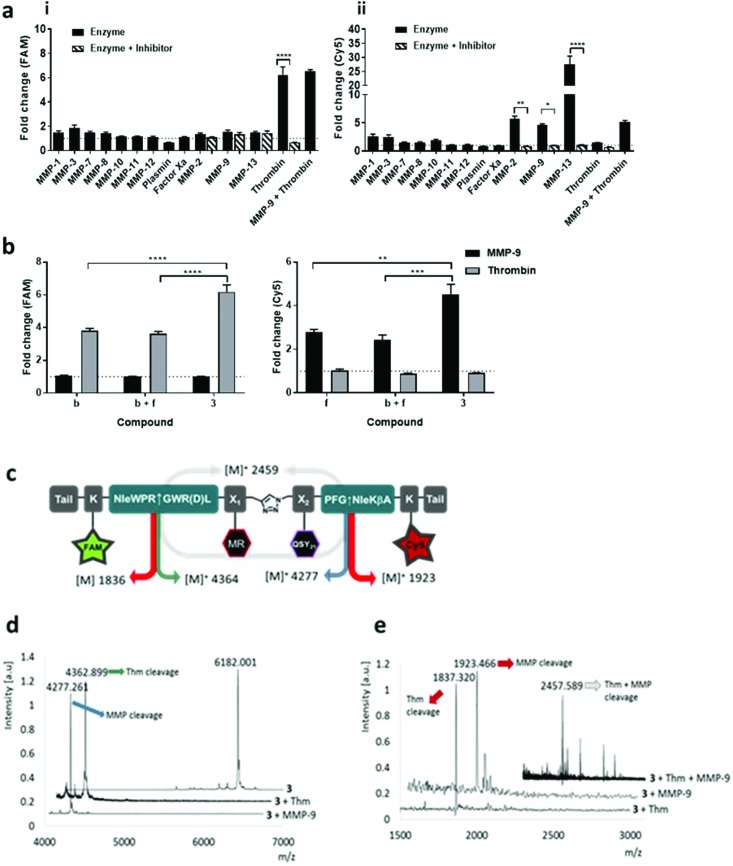
Dual-probe **3** was cleaved by target (i) thrombin and (ii) MMPs, specificity was confirmed by MALDI TOF MS. (a) Dual-probe **3** was incubated with target and off-target enzymes, with or without inhibitor. Activation was determined by measuring the fluorescence increase (compared to enzyme-free control) at 10 min at two different wavelengths (ex/em 485/528 nm (FAM) and 640/670 nm (Cy5)). (b) The fold-change in fluorescence intensity of fragments **b**, **f** and dual-probe **3** were compared following activation with MMP-9 or thrombin, with probe **3** showing specific FAM activation with thrombin and Cy5 increasing with MMP-9 (c) structure of dual-probe **3** showing the theoretical molecular weights of the fragments obtained following cleavage; (d) MALDI-TOF MS spectra of **3** before and after treatment with thrombin or MMP-9. The initial peak for **3** at *m*/*z* 6182.001 Da (calc. for C_307_H_427_N_64_O_67_S_3_^+^ [M^+^]: 6182.353 Da) disappears following treatment showing two new peaks with the expected *m*/*z*. (e) MALDI-TOF MS (*m*/*z* 1500–3000 Da) correlates well with the expected fragments even when the two enzymes act together. Data is the mean of three independent replicates performed in duplicate. Error bars represent s.e.m. Statistical analysis was performed with one-way ANOVA test. (a) * *P* = 0.0225; ** *P* = 0.0026; **** *P* < 0.0001. (b) P** = 0.0018; *** *P* = 0.0004; **** *P* < 0.0001.

The two lead optimized FRET peptides for thrombin and MMP described above (fragments **b** and **f**, Table S2†) were separately synthesized by Fmoc solid-phase peptide chemistry (Scheme S1†). The dyes and quenchers were coupled onto the peptides by solid-phase methods, with the Dde^32^ group used to allow late stage fluorophore incorporation, whilst the quenchers were added onto the amino termini of each peptide. In both cases C-terminal spacers consisting of bis-PEG units and (d)-Lys were incorporated; providing a hydrophilic unit to promote solubility and preventing exopeptidase degradation, and again, were incorporated as a result of an extensive screening study (see details in the ESI†) to stop degradation *via* enzymes present in homogenized human tissue. Following cleavage from the resin, the two peptides were purified and azide/alkyne coupling of the two unprotected fragments was achieved under aqueous conditions,^33^ allowing efficient conjugation in 5 hours *via* generation of a biocompatible 1,2,3-triazole (Fig. 1ii and iii), and giving compounds **1–3** (Fig. 1, Table 1). Importantly, combining fragments **b** and **f** onto one scaffold to give dual-probe **3** provided enhanced quenching of each of the fluorophores (Fig. 1iv) and showed increased dequenching and improved signal-to-noise upon proteolytic cleavage compared to the individual fragments (Fig. 2). Compounds **4** and **5** were prepared incorporating d-aminoacids into the cleavage site for each enzyme to provide non-cleavable control compounds (Fig. 1, Table 1). The final constructs have molecular weights of 6.1–6.4 kDa and represent highly refined, sophisticated, selective and sensitive optical probes.

The enzymatic cleavage of the dual-probe was specific; with MALDI-TOF MS confirming cleavage between –Gly↑Nle by MMPs and between Arg↑Gly by thrombin (see Fig. 2). This was also shown by HPLC analysis (Fig. S3b†). Activation of dual-probe **3** was prevented by marimastat (a pan-MMP inhibitor) and anti-thrombin III (AT3) (Fig. 2). Control dual-probes **4** and **5** (Table 1) were not activated by their target enzymes, confirming enzyme specificity (Fig. S4†).

Dual-probe **3** was stable for over two months in PBS at room temperature (Fig. S2†), optically silent prior to activation (Fig. S3a†) and completely plasmin and Factor Xa resistant, whilst retaining its ability to be activated by target enzymes – showing high signal to noise (Fig. 2a). Within 10 minutes of enzyme addition to dual-probe **3**, Cy5 signal increased 5.6, 4.6 and 27.4-fold by the active domains of MMP-2, -9 and -13 respectively, while the FAM signal increased 6.2-fold following the addition of thrombin with the fold-changes in fluorescence superior to those of individual thrombin and MMP compounds **b** and **f** (Fig. 2b). Vitally, no signal increase was observed as a consequence of cross-enzyme reactivity, and no steric hindrance was seen when cleaving the dual-probe with MMP and thrombin either simultaneously (Fig. 2a), or sequentially (Fig. S3c†).

### Human tissue validation

**Fig. 3:**
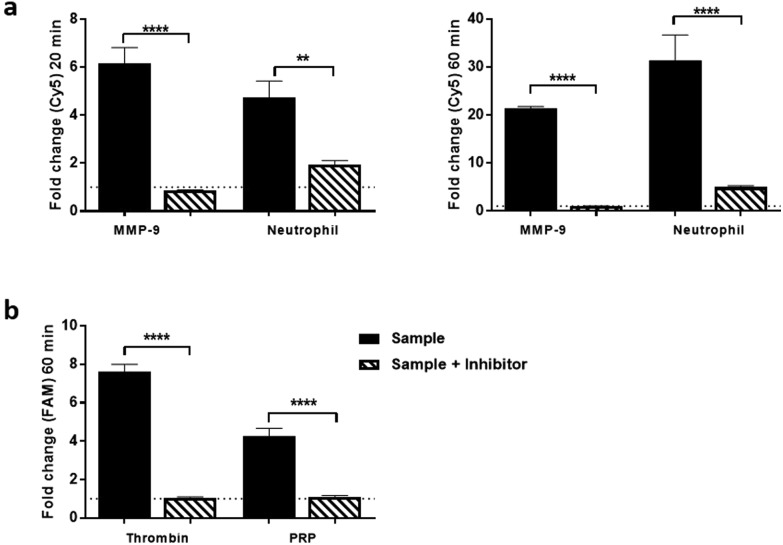
Dual-probe **3** was cleaved selectively by: (a) activated neutrophil supernatant and (b) Platelet rich plasma (PRP). Cleavage and subsequent activation was determined by measuring the relative fluorescence increase (compared to enzyme-free control). Data shows mean of three independent replicates performed in duplicate ((a) 20 and 60 min time points for neutrophil assay, (b) 60 min time point for PRP assay). Error bars represent s.e.m. Statistical analysis was performed with one-way ANOVA test, enzyme columns were compared directly to enzyme plus inhibitor conditions. ** *P* < 0.01; *** *P* < 0.0001; **** *P* < 0.0001.

Dual-probe **3** was subsequently evaluated in escalating *ex vivo* human primary tissue models to demonstrate stability within complex microenvironments and activation of the dual-probe with biologically relevant levels of target enzyme. This included activated primary isolated human neutrophils, primary human platelet rich plasma (PRP) (Fig. 3 and S5, outlined in ESI†), human atherosclerotic plaque and finally to show proof of concept, human lung adenocarcinoma samples.

**Fig. 4:**
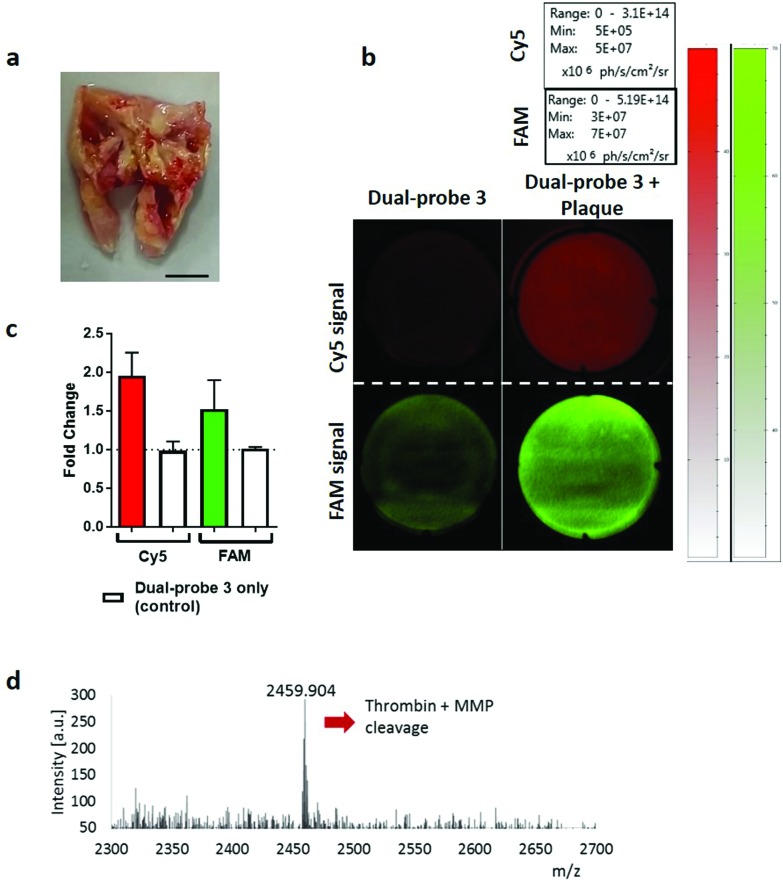
Dual-probe **3** was specifically activated by excised atherosclerotic plaques. (a) Representative image of excised atherosclerotic plaque. Scale bar 5 mm. (b) Cleavage and subsequent activation of dual-probe **3** by atherosclerotic tissue was determined by measuring relative fluorescence increase at ex/em 485/528 nm (FAM) and 640/670 nm (Cy5) over 60 min. (c) Fold-change in fluorescence over 60 min. Data is the mean of three independent samples. Error bars represent s.e.m. (d) MALDI-TOF MS analysis was performed after incubation with the atherosclerotic plaque. The peak at 2459.9 Da indicates combined thrombin and MMP cleavage (Fig. 2 and Fig. S6† for further detail).

Human atherosclerotic plaques have a biologically complex microenvironment, with MMPs and thrombin in high abundance.^34^,^35^ These were thus used as a relevant non-cancer model to validate dual-probe **3** (Fig. 4). Activation of the dual-probe by the endogenous enzymes was measured by fluorescence imaging of the supernatant after 60 min (Fig. 4b). Fluorescence emitted by dual-probe **3** incubated with tissue samples increased in both the FAM and Cy5 channels (Fig. 4c) with MALDI-TOF MS analysis confirming that activation was specific, with fragments of 1837 Da (thrombin cleavage) and 2459 Da (thrombin + MMP cleavage) detected (Fig. 4d, Fig. S6†). Where no tissue was present, only intact probe was identified by MALDI-TOF MS and no increase in fluorescence was detected. Whilst fold-changes in fluorescent signal were observed to be modest when imaging with this widefield technique (reasons for which are outlined in the ESI-Fig. S6†), activated dual-probe **3** from these clinical samples was detected by both optical imaging and MALDI-TOF MS analysis.

**Fig. 5:**
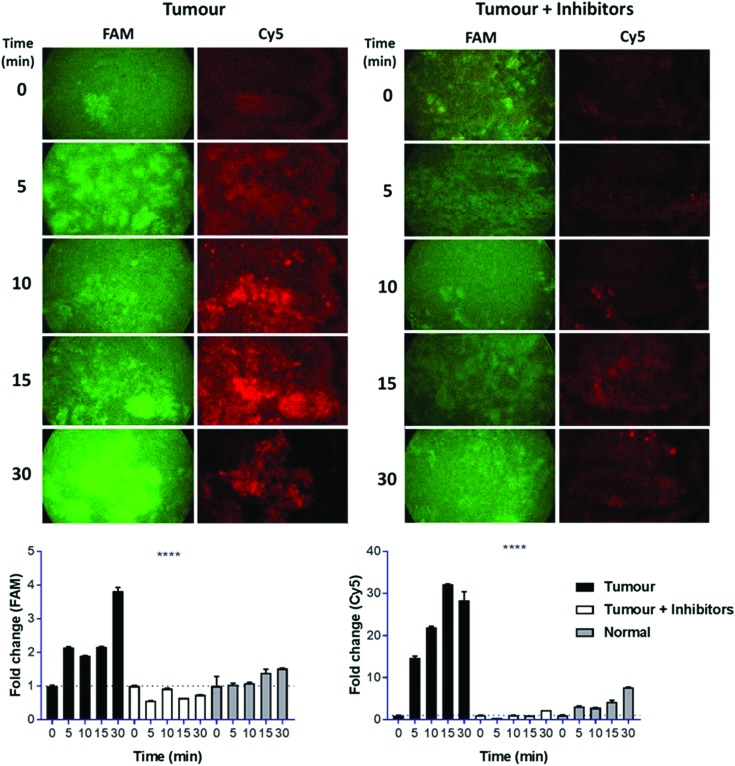
Specific activation of dual-probe **3** was measured in human lung adenocarcinoma with a clinical optical fibre-based imaging device, ± inhibitors. Average fold-change in fluorescence for each tissue type was calculated for each time point for the FAM and Cy5 signal. Images show representative single frames from the imaging system, while graphs show calculated fold-change in average fluorescence. Error bars show s.e.m, **** *p* < 0.0001. Statistical analysis was performed with one-way ANOVA test.

An initial clinical use of dual-probe **3** could be in the rapid identification of fibroproliferative enzymes around human lung adenocarcinoma *in situ*; where the dual-probe could be locally delivered and immediately imaged by a multi-colour fibre-based imaging system during an interventional procedure such as bronchoscopy or image-guided biopsies.^30^ Typically pulmonary microendoscopy of suspected lung cancer is hampered by the lack of molecular tracers to augment tissue autofluorescence signatures.^5^ Combining local intrapulmonary delivery of dual-probe **3** with multi-colour microendoscopy could significantly enhance clinical diagnostic capability. Within 5 min of dual-probe **3** addition to human lung adenocarcinoma tissue *ex vivo*, a 2.1 and 14.7-fold increase in FAM (thrombin) and Cy5 (MMP-13, -9, -2) signal was measured using a clinically approved fibre-based imaging system.^30^ The fold-change increased to 3.8 and 32.2-fold at 30 min (Fig. 5). The increase in fluorescence was not apparent when thrombin and MMP inhibitors were added to the tissue, and fold-changes from native tumour tissue were significantly elevated compared to those measured from the dual-probe on ‘healthy’ lung tissue (*p* < 0.0001), which was collected from the same patient, but from an area distinct from the tumour. Thus supporting the use of dual-probe **3** to highlight aberrant matrix activity as an aid in guiding biopsy and in ‘red flagging’ a lesion as suspicious; a major advancement in the field of pulmonary endomicroscopy, required as we head towards a future of ‘optical biopsy’ and guided interventional procedures.^5^,^36^

## Conclusions

In summary, we have developed a novel FRET-based dual-probe that can simultaneously detect human thrombin and MMP activity, with reporting at two spectrally distinct wavelengths. It was synthesised as two component halves and efficiently brought together using azide/alkyne cycloaddition chemistry to give a sophisticated synthetic dual imaging probe. This dual-probe shows long-term stability, and despite its size and complexity, the optimized synthetic strategy is practical and readily scalable. The dual-probe was specifically designed for human clinical translation and was not activated by murine proteolytic enzymes; as evidenced by using lavage supernatants from murine models and using murine platelet rich plasma (see Fig. S7†), highlighting the significant caveats of using murine models as experimental systems to support human translation.

The probe exhibits an increase in florescence upon activation by target MMPs and thrombin which was detectable by all currently available detection systems used in the experimental evaluation. However, there are potential challenges as with any biosensor in achieving an appropriate limit of detection (LoD) *in vivo*. *In vivo in situ*, the LoD is unknown and likely to be highly variable regarding expression of enzyme activity, and to date, non-quantitative immunohistochemistry and gene expression have been used in lung cancers to quantify presence rather than kinetic enzyme determination. However, in non-small-cell lung carcinoma, MMP13 has been shown to be consistently and ubiquitously expressed.^37^ Ultimately the LoD will need to be evaluated in formal prospective diagnostic studies, however excitingly, with more sensitive detectors that are capable of measurements independent of fluorescent intensity that are now emerging,^38^ we expect this to be ample for detection.

The incubation of dual-probe **3** within escalating human relevant environments described here demonstrates that the dual-probe possessed excellent stability, specificity and underwent highly selective activation. Due to the superior stability, specificity and signal-to-noise of our novel dual-probe compared to those reported within the literature, we believe that this dual-probe has potential to be used as a tool to identify multiple biomarkers of the fibroproliferative pathway in fluid and tissue samples *ex vivo*, but more importantly in the future with further translational development, *in situ in vivo* optical imaging. In addition, the platform approach lends itself to versatile insertion of other peptide substrates to generate future multiplexed chemosensors of enzyme activity.

## Conflicts of interest

There are no conflicts to declare.

## Supplementary Material

Supplementary information
