# Supplementary material for: Bimodal fluorogenic sensing of matrix proteolytic signatures in lung cancer
Source: Org Biomol Chem. 2018 Aug 23;16(43):8056–63. doi: 10.1039/c8ob01790e (PMC6238727; doi:10.1039/c8ob01790e)
Supplement: Supplementary file 1 [file OB-016-C8OB01790E-s001.pdf]

Electronic Supplementary Material (ESI) for Organic & Biomolecular Chemistry.
This journal is © The Royal Society of Chemistry 2018

SUPPLEMENTARY INFORMATION

BIMODAL FLUOROGENIC SENSING OF MATRIX PROTEOLYTIC SIGNATURES IN LUNG CANCER

Alicia Megia-Fernandez[a#], Bethany Mills[b#], Chesney Michels[b], Sunay V. Chankeshwara[a], Nikola Krstajić[c], Chris Haslett[b], Kevin Dhaliwal*[b] & Mark Bradley*[a]

[a]School of Chemistry and the EPSRC IRC Proteus, University of Edinburgh, Joseph Black Building, David Brewster Road, Edinburgh, EH9 3FJ, UK.

[b]MRC Centre of Inflammation Research, Queen's Medical Research Institute, University of Edinburgh, 47 Little France Crescent, EH16 4TJ, Edinburgh, UK.

[c]Institute for Integrated Micro and Nano Systems, School of Engineering, University of Edinburgh, EH9 3JL, UK.

[#]Authors contributed equally.

*E-mail: Mark.Bradley@ed.ac.uk and Kev.Dhaliwal@ed.ac.uk

<u>Contents:</u>

- Supplementary Data (Figures S1-S7)
- Materials and Methods (Chemistry)
- Materials and methods (Biology)
- Peptide sequence optimization of the MMP-2/9/13 substrate
- References

SUPPLEMENTARY DATA

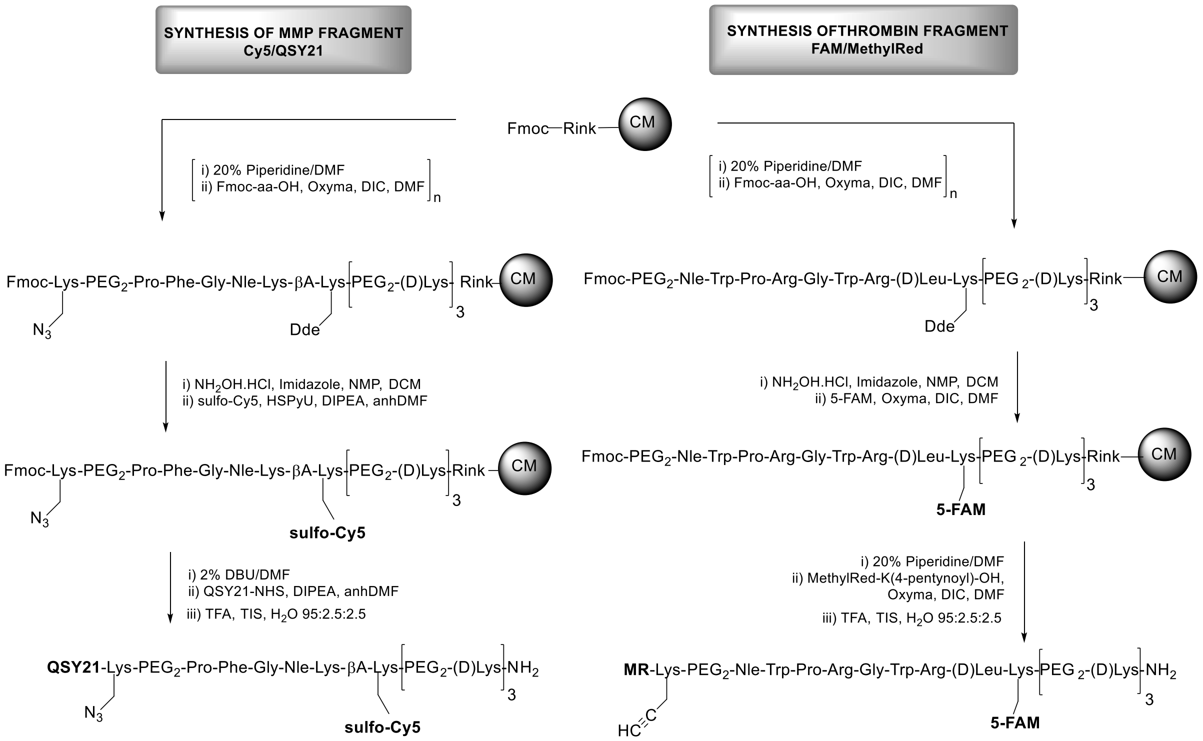

Compound f **Compound b**

Scheme S1. Synthetic route to the two fragments of the dual-probe **3** by SPPS. *For clarity the acid labile protecting groups of the amino acids have been omitted. CM = Chemmatrix™ resin

Left: MMP cleavable peptide containing the azide, Sulfo-Cy5 and QSY21 groups (compound **f**).

Right: Thrombin cleavable peptide containing the alkyne, Methyl Red (MR) and 5-Carboxyfluorescein (5-FAM) groups (compound **b**).

Full details to the synthetic routes are provided in the Materials and Methods section below.

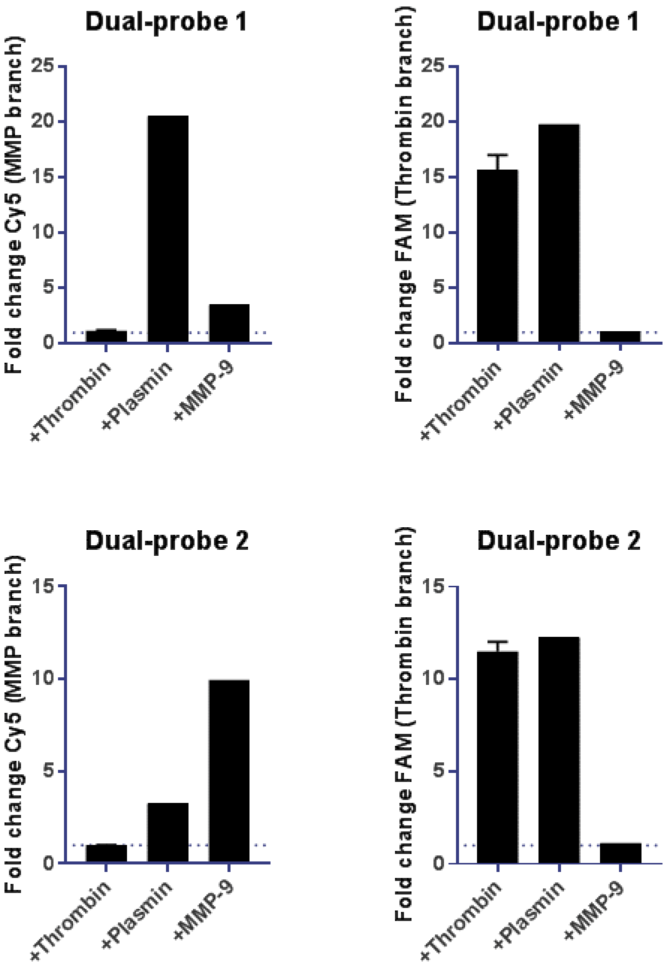

Figure S1. Enzyme specificity of the 1st and 2nd generation dual-probes: Data shows the fold change in fluorescence over background provided by dual-probes **1** and **2** with Thrombin, MMP-9 and Plasmin after 10 minutes using a multi-well plate fluorimeter with excitation/emission 485/528 nm (FAM, thrombin branch) and 640/670 nm (Cy5, MMP branch).

Top left: The dual probe 1 was analysed for increase in Cy5 intensity generated by cleavage of the MMP-sequence. Note the huge cleavage caused by Plasmin.

Top right: The dual probe 1 was analysed for increase in FAM intensity generated by activation of the Thrombin-based sequence. Note cleavage by both Thrombin and Plasmin.

Bottom left: The dual probe 2 showed much better cleavage by MMP than Plasmin.

Bottom right: The dual probe 2 was still cleaved by Thrombin and Plasmin.

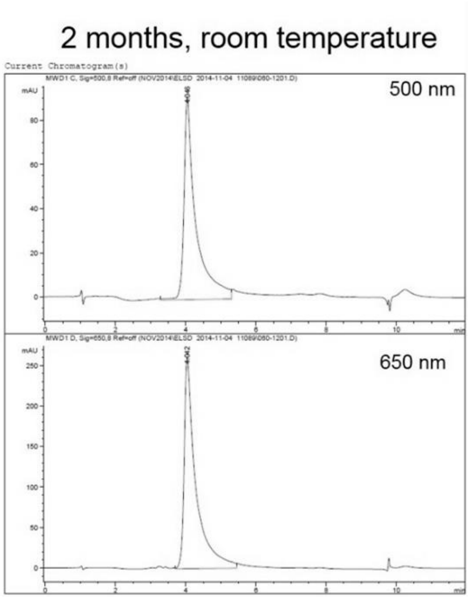

Figure S2. Stability data: HPLC trace of compound **3** after 2 months in PBS at room temperature with detection at 500 and 650 nm.

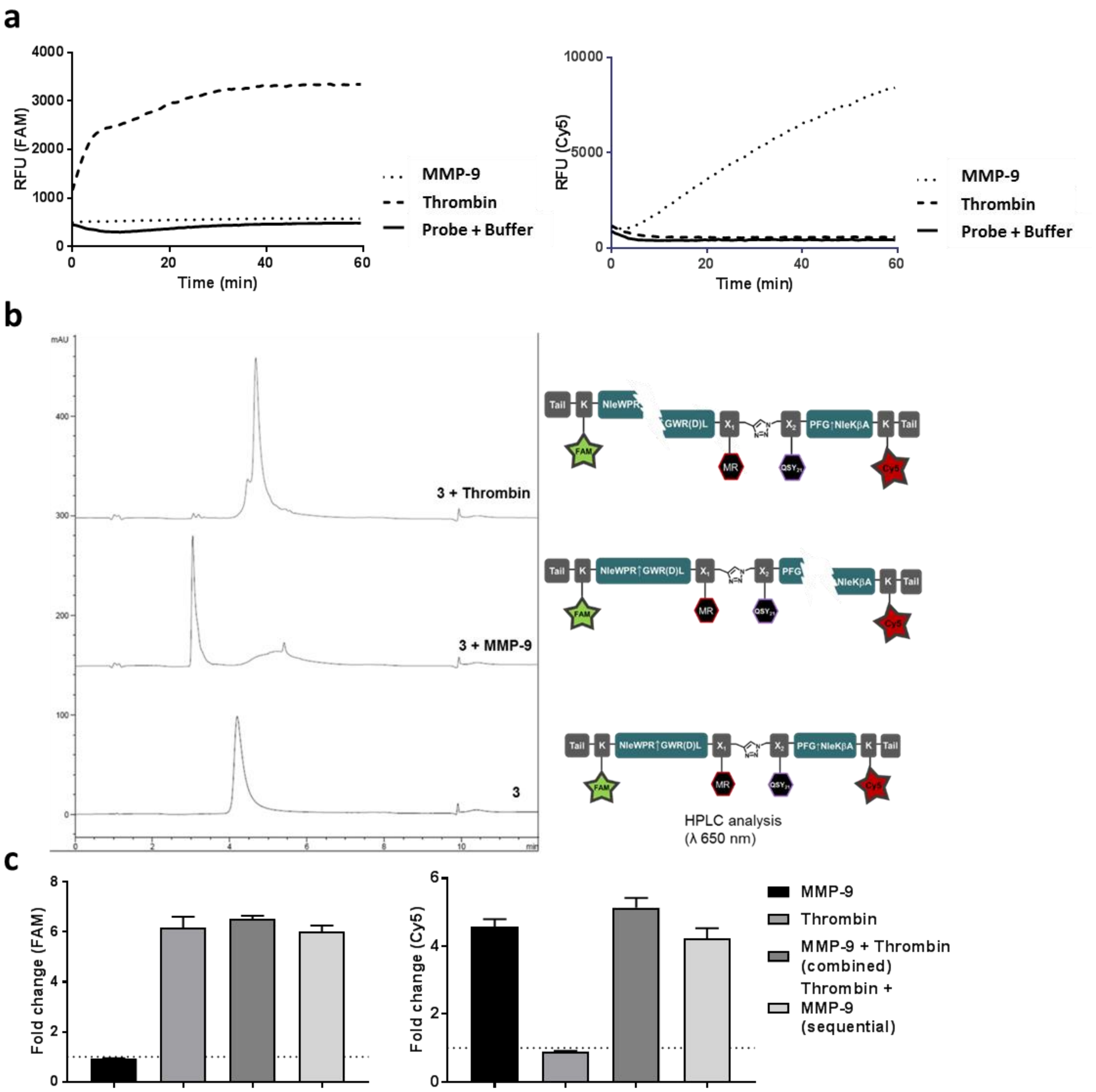

Figure S3. Dual-probe 3 is optically quiet in its native state with specific cleavage demonstrated by HPLC analysis. Dual-probe **3** was incubated with MMP-9, thrombin or buffer. Cleavage and subsequent activation of the probe were determined by: (**a**) fluorescence (relative fluorescence units, RFU) with ex/em 485/528 (FAM), 640/670 (Cy5) and (**b**) HPLC analysis with absorbance detection at 650 nm before (lower trace) and after treatment with MMP-9 (middle trace) or Thrombin (upper trace). After MMP-9 cleavage the fragment containing Cy5 increased in polarity, while after thrombin cleavage the fragment containing Cy5 decreased in polarity and the retention time was slightly longer. (**c**) Dual probe **3** incubated with either MMP-9, Thrombin or both (either combined or added sequentially). Fold changes were calculated from enzyme free controls. Where enzymes were added sequentially, initial incubations with the first enzyme were for 60 minutes. Data is plotted after 10 minutes for each condition/combination or after the second enzyme is added. [(FAM) ex/em 485/528, (Cy5) ex/em 640/670]. Error bars show s.e.m.

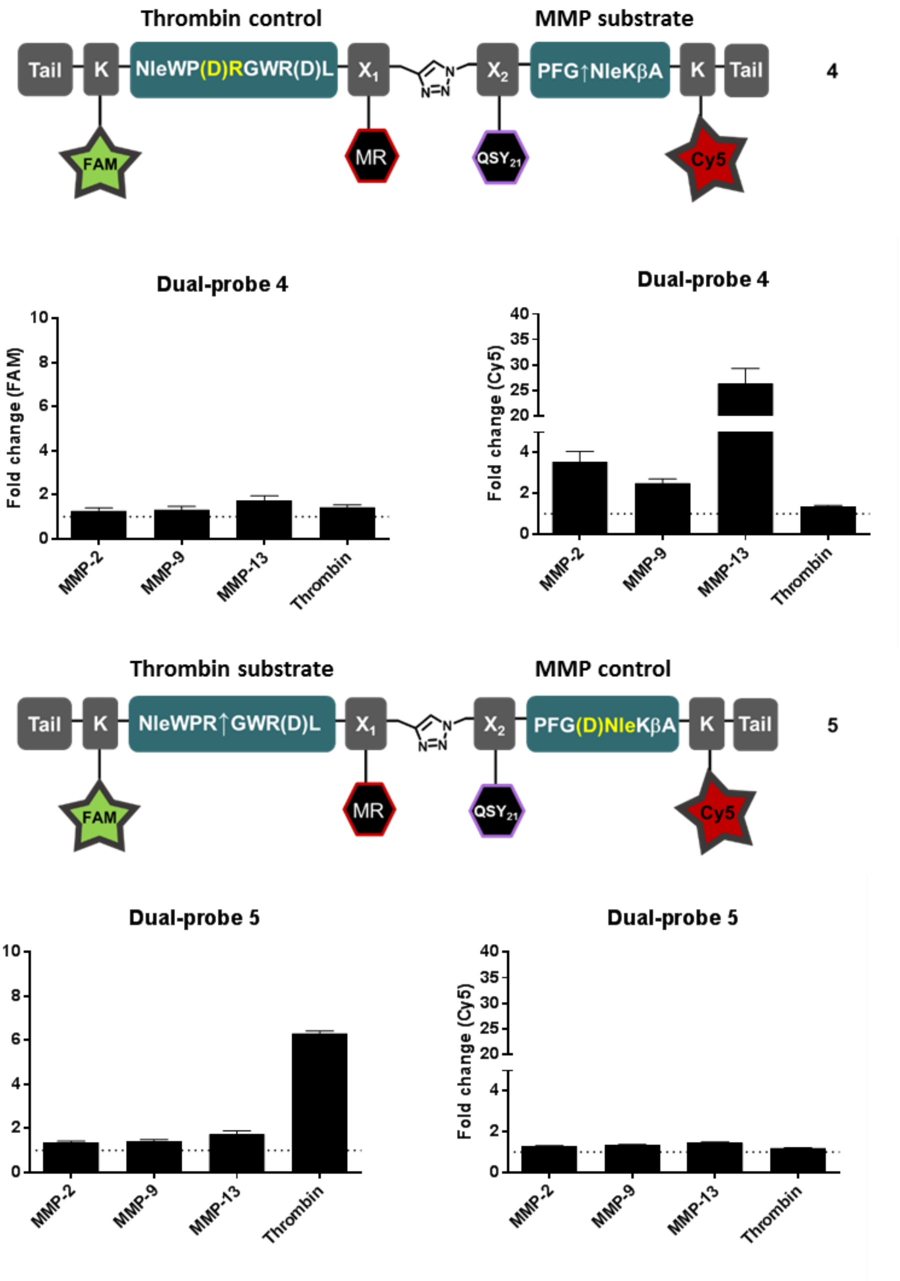

Figure S4. Substitution of L-amino acids for D-amino acids in the peptide cleavage sites prevents probe activation. Specificity of each target peptide sequence for MMP and thrombin (Thm) was confirmed by positioning a D-amino acid into the cleavage site of the thrombin branch (control dual-probe **4**) and the MMP branch (control dual-probe **5**) and incubation with MMP or thrombin. Fold changes [(FAM) ex/em 485/528, (Cy5) ex/em 640/670] refer to relative change in fluorescence intensity compared to enzyme-free controls at 10 min.

Dual-probe 3 was cleaved by activated neutrophil supernatant *ex vivo*

Upon degranulation of activated human neutrophils, a plethora of cytokines, reactive oxygen species and antimicrobial peptides[1] as well as proMMPs and human neutrophil elastase (HNE)[2] are released into the extracellular environment. HNE is able to cleave proMMP-9 into its active form[3]. Dual-probe **3** (+/- 20 µM marimastat) was incubated with supernatants from freshly isolated/activated neutrophils. The Cy5 signal from dual-probe **3** was elevated 4.7-fold within 20 minutes of probe addition and was increased by 26-fold at 60 min (Fig. 3a, Fig. S5), comparable to that achieved by recombinant MMP-9 (6.2-fold within 20 min and 21-fold by 60 min). Cy5 signal elevation by activated neutrophil supernatant was significantly reduced in the presence of marimastat (**P = 0.0019) with just a 1.9-fold increase observed after 20 mins (identical to that observed with non-activated neutrophils) (Fig. S5a). No increase in FAM signal was observed under any of these conditions (Fig. S5a).

Dual-probe 3 was cleaved by thrombin activity within platelet rich plasma *ex vivo*

Platelet rich plasma (PRP) was utilised as a complex biologically relevant source of thrombin. Dual-probe **3** was incubated with PRP extracted from healthy whole human blood. Due to a lag phase before thrombin activation[4] no cleavage of dual-probe **3** by thrombin in PRP was initially observed (Fig. S5b), however by 60 min, FAM signal was elevated 4.3 fold (compared to 7.6-fold increase for purified thrombin) (Fig. 3b). The activation of dual-probe **3** was completely inhibited by the presence of AT3 for both thrombin and the PRP conditions (P< 0.0001). No activation of the Cy5 branch was observed after 60 minutes (Fig. S5c).

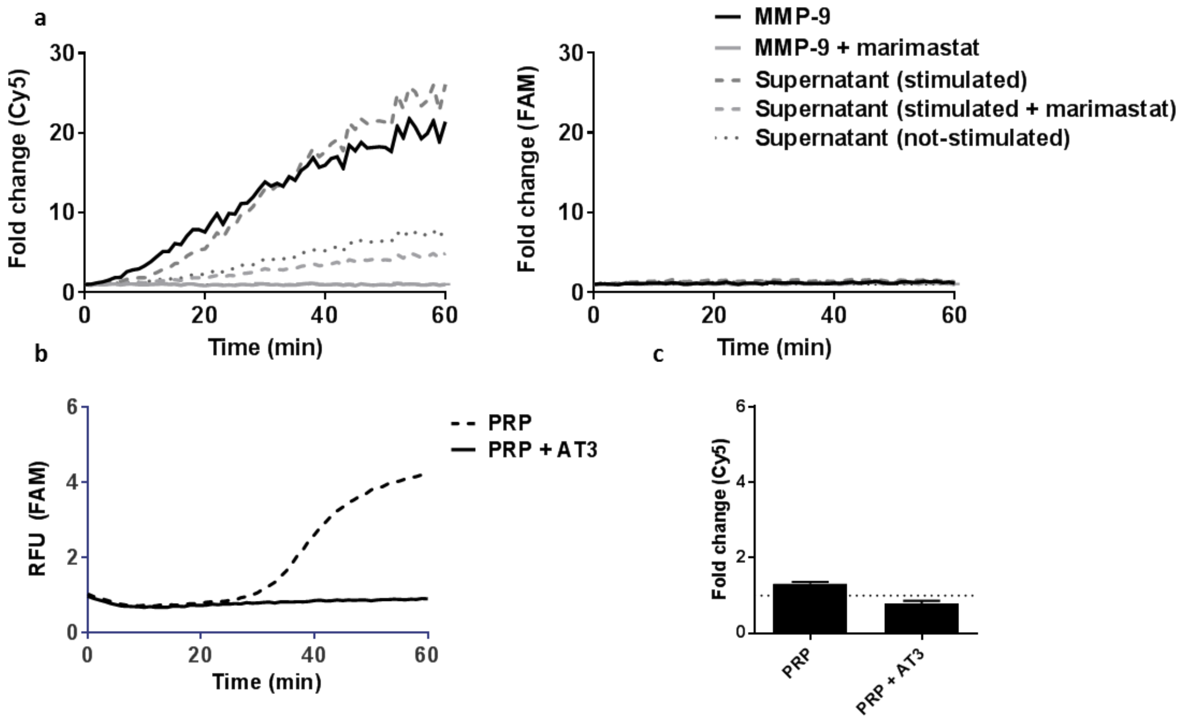

Figure S5. Dual-probe 3 was specifically activated within complex biological samples.

(**a**) Activation of dual-probe **3** by supernatants from stimulated and non-stimulated neutrophils over 60 min. The MMP inhibitor marimastat was pre-incubated with supernatants collected from stimulated neutrophils. Increase in Cy5 and FAM signal was measured. Data shows the mean of two independent replicates performed in duplicate. (**b**) Dual-probe **3** was activated by platelet rich plasma (PRP). This activation was quenched when the thrombin inhibitor anti-thrombin III (AT3) was pre-incubated with the PRP. (**c**) No off-target activation of the MMP branch was measured after 60 min with the PRP. Data shows mean of three independent replicates performed in duplicate. Error bars represent s.e.m

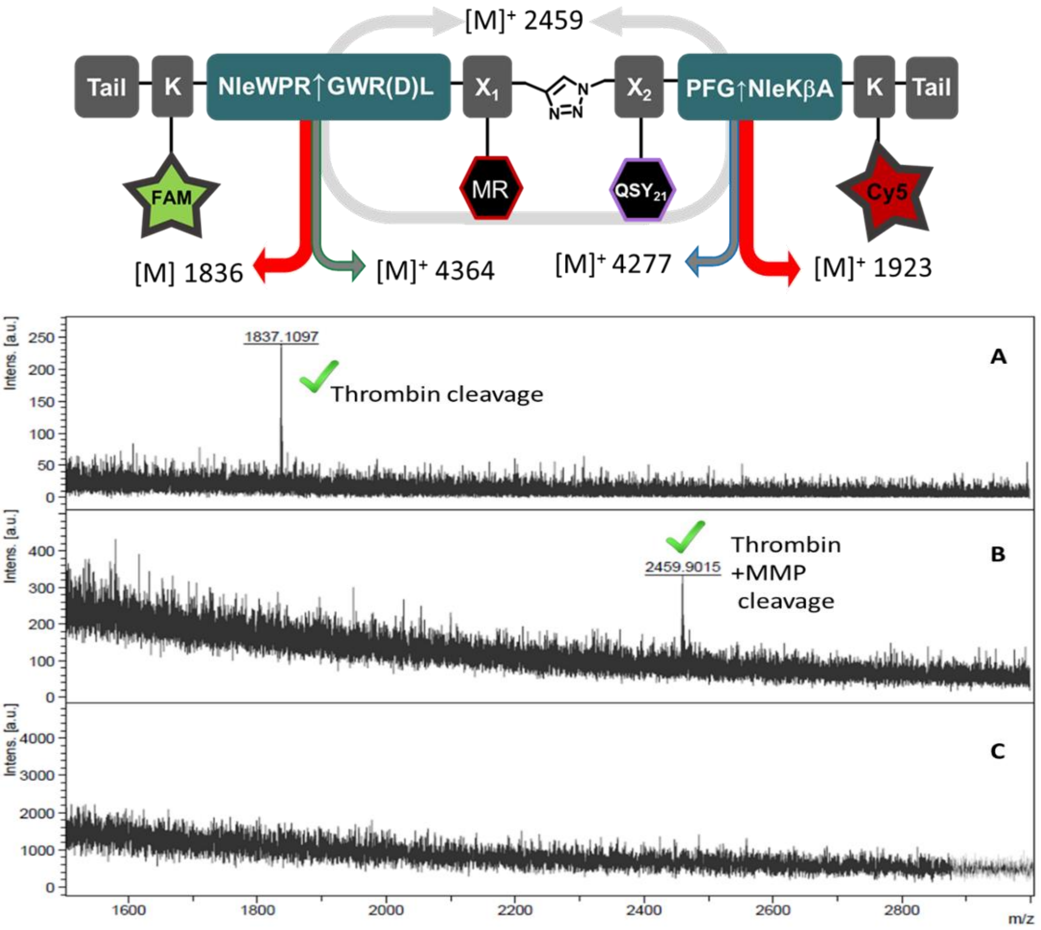

Figure S6. MALDI-TOF MS analysis confirmed the specific cleavage of dual-probe 3 following enzymatic reaction with human carotid samples. (**A**) Samples analysed directly by MALDI-TOF MS showing a peak at m/z 1837.1097 (calc. for $C_{88}H_{131}N_{20}O_{23}$ $[M+H]^+$: 1837.1325) corresponding to one of the fragments caused by thrombin cleavage. (**B**) Samples analysed after ZipTip™ (Millipore) treatment showing a peak at m/z 2459.9015 (calc. for $C_{129}H_{158}N_{25}O_{23}S^+$ $[M]^+$: 2458.8945) corresponding to the central fragment after thrombin and MMP cleavage. (**C**) Control sample (untreated).

Whilst fold-changes in fluorescent signal were observed to be modest by imaging with this technique (Fig 4), negative controls did not increase in fluorescence and specificity of probe cleavage by MMPs and thrombin was confirmed by MALDI-TOF MS analysis. The global enzymatic levels on the surface of the plaque could be limited for a number of reasons. The extent of plaque in each surgical resection was varied, and it would not be expected that the whole of the excised tissue would be highly active in MMP. Additionally, washing of the atherosclerotic plaque in PBS was necessary following surgical extraction, and this would likely have removed enzyme. Despite this, activated dual-probe **3** was detected by imaging and MALDI-TOF MS.

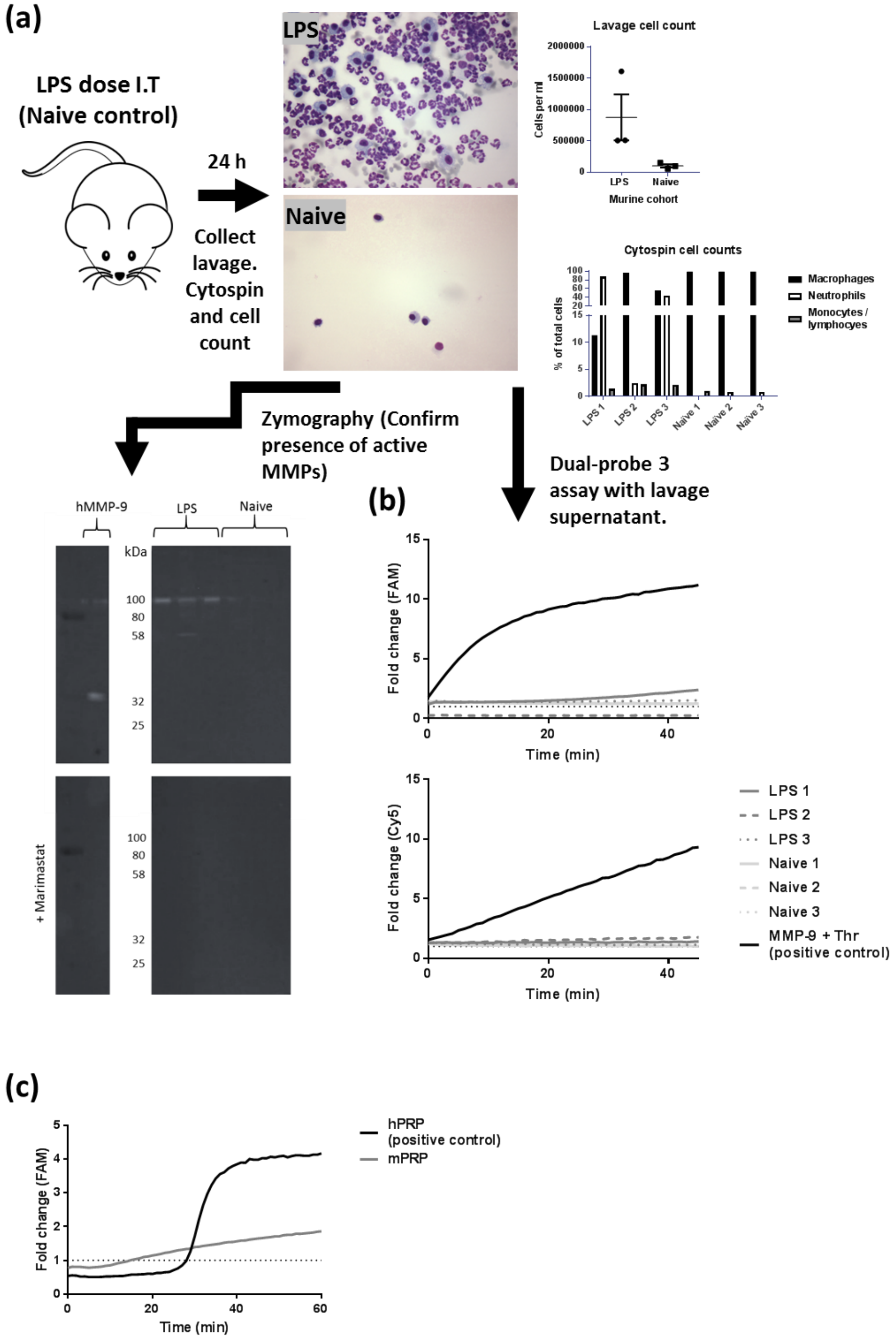

Figure S7. Dual probe 3 was not cleaved by murine proteolytic (MMP and Thrombin) enzymes. **(a)** LPS treatment (by intratracheal route) to murine lungs causes inflammation, with massive cellular increases, primarily by neutrophils, as shown in the cytospins (top), compared to naïve controls. Active MMP (murine MMP-9, 92 kDa) was present in all lavage samples from LPS treated mice, confirmed by gelatin based zymography. This was not present in the lavage from naïve mice. **(b, c)** Dual-probe **3** was incubated with the lavage fluid and platelet rich plasma (PRP) collected from the LPS murine model, and with control human MMP-9 and thrombin. Cleavage and subsequent activation was determined by measuring the relative fluorescence increase (compared to buffer only control) [ex/em 485/528 nm (FAM) and 640/670 nm (Cy5)].

MATERIALS AND METHODS (CHEMISTRY)

1. <u>General :</u>

Commercially available reagents were used without further purification. Methyl Red and 4-pentynoic acid were purchased from Sigma, 5-Carboxyfluorescein was purchased from Carbosynth. Ltd. Fmoc-Lys(N_3)-OH, [2-[2-(Fmoc-amino)ethoxy]ethoxy]acetic acid and 2-((2-azidoethoxy)ethoxy)acetic acid were purchased from Iris Biotech. QSY21-NHS ester was prepared according to a previously reported procedure.[5] NMR spectra were recorded using Bruker AC spectrometers operating at 500MHz for 1H. Chemical shifts are reported on the δ scale in ppm and are referenced to residual non-deuterated solvent resonances in deuterated solvents. Normal phase purifications by column chromatography were carried out on silica gel 60 (230-400 mesh). Analytical reverse-phase high-performance liquid chromatography (RP–HPLC) was performed on an Agilent 1100 system equipped with a Discovery C18 reverse-phase column (5 cm x 4.6 mm, 5 µm) with a flow rate of 1 mL/min and eluting with $H_2O/CH_3CN/HCOOH$ (95/5/0.1) to $H_2O/CH_3CN/HCOOH$ (5/95/0.1), over 6 min, holding at 95% ACN for 2 min, with detection at 254, 500 and 650 nm and by evaporative light scattering. Semi-preparative RP–HPLC was performed on an Agilent 1100 system equipped with a Zorbax Eclipse XDB-C18 reverse-phase column (250 x 9.4 mm, 5 µm) with a flow rate 2.0 mL/min and eluting with 0.1% HCOOH in H_2O (A) and 0.1% HCOOH in CH_3CN (B), with a gradient of 5 to 95% B over 30 min and additional isocratic period of 5 min. Electrospray ionization mass spectrometry (ESI–MS) analyses were carried out on an Agilent Technologies LC/MSD Series 1100 quadrupole mass spectrometer (QMS) in an ESI mode. MALDI spectra were acquired on a Bruker Ultraflextreme MALDI TOF/TOF with a matrix solution of sinapic acid (10 mg/mL) in H_2O/CH_3CN/TFA (50/50/0.1). ZipTip™ C-18 (Millipore, MA) pipette tips were used to analyse samples after tissue treatment by MALDI.

<u>2. Probe synthesis. General methods</u>

The FRET peptide sequences for MMP and thrombin were individually synthesized by standard Fmoc solid-phase peptide chemistry. Dyes and quenchers were coupled also by standard solid-phase methods. General procedures are as follows:

Manual peptide synthesis was performed on Aminomethyl-ChemMatrix™ resin using an Fmoc-Rink amide linker.

Coupling of Fmoc-Rink amide linker: The Fmoc-Rink-amide linker (0.54g, 1.0 eq) was dissolved in DMF (10 mL) and Oxyma (0.14g, 1.0 eq.) was added and the mixture was stirred for 10 min. Diisopropylcarbodiimide (DIC, 155 µL, 1.0 eq.) was then added and the solution stirred for 1 min before adding it to Aminomethyl-ChemMatrix resin (1.0 g, 1.0 mmol/g). The resulting mixture was stirred at 50^0C for 45 min and washed with DMF (3x10 mL), DCM (3x10 mL) and MeOH (3x10 mL). Finally the resin was treated with Ac_2O:Py:DMF (2:3:15) for 30 min in order to cap any remaining free amino groups and it was washed again with DMF (3x10 mL), DCM (3x10 mL) and MeOH (3x10 mL). Resin loading[6] was measured as ~0.58 mmol/g.

Fmoc deprotection: In general, to the resin pre-swollen in DCM was added 20% piperidine in DMF and shaken (2x10 min). The solution was drained and the resin washed with DMF (3x10 mL), DCM (3x10 mL) and MeOH (3x10 mL). In the cases were Fmoc deprotection was carried out on Cy5 containing peptides, a solution of 2% DBU in DMF (2 × 10 min) was used.

Aminoacid coupling: A solution of the appropriate D- or L-amino acid (3.0 eq per amine) and Oxyma (3.0 eq) in DMF (0.1M) was stirred for 10 min. DIC (3.0 eq) was added and stirred for 1 min. The pre-activated mixture was then added to the resin pre-swollen in DCM and the reaction heated at 50^0C for 30 min. The solution was drained and washed with DMF (3x10 mL), DCM (3x10 mL) and MeOH (3x10 mL). Completion of coupling reactions were monitored by a Kaiser test or Chloranil test (when secondary amines are involved). The side chain protecting group used were Boc for Arginine, Tryptophan and Lysine. Fmoc-Lys(Dde)-OH was used as orthogonal reagent to introduce the dyes.

Coupling of other carboxylic acids: Coupling of {2-[2-(Fmoc-amino)ethoxy]ethoxy}acetic acid (PEG_2), 5-Carboxyfluorescein (FAM), Fmoc-Lys(N_3)-OH and Methyl Red-Lys-(4-pentynoyl)-OH was carried out following the procedure described for the aminoacid coupling.

Dde deprotection: (a) Dde deprotection in non Fmoc-containing peptides was carried out as follows: To the resin pre-swollen in DCM was added 2% hydrazine in DMF and shaken (5x10 min). The solution was drained and the resin washed with DMF (3x10 mL), DCM (3x10 mL) and MeOH (3x10 mL). (b) Selective Dde deprotection[7] in Fmoc-protected peptides was achieved with a solution containing Imidazole (1.35 mmol) and Hydroxylamine hydrochloride (1.80 mmol) in NMP (5 mL). After complete dissolution 5 volumes of this solution were diluted with 1 volume of CH_2Cl_2 and the resin was treated with the final mixture for 3h at room temperature. The solution was drained and the resin washed with DMF (3x10 mL), DCM (3x10 mL) and MeOH (3x10 mL).

Sulfo-Cy5 dye coupling: A solution containing sulfo-Cy5 (1.0 eq per amine) in anhydrous DMF (10 mg/mL) was activated with N,N,N',N'-Bis(tetramethylene)-O-(N-succinimidyl)uronium hexafluorophosphate (HSPyU) (1.0 eq) and DIPEA (3 eq) at 40^0C for 1h. Once the activation was complete the solution was added to the resin together with DIPEA (3 eq) and shaken overnight. The solution was drained and the resin washed with DMF until colourless wash solution, then DCM (3x5 mL) and MeOH (3x5 mL).

QSY21 coupling: N-terminal capping with QSY21-NHS ester (1.0 eq per amine) was carried out in anhydrous DMF (0.1M) containing DIPEA (3 eq) for 12 h. The solution was drained and the resin washed with DMF until the wash solution was colourless, then DCM (3x5 mL), MeOH (3x5 mL) and finally ether (3x5 mL).

Methyl Red-NHS coupling: Methyl Red-NHS ester (1 eq) coupling on the solid-phase was carried out in anhydrous DMF (0.1M) containing DIPEA (3 eq) for 12 h. The solution was drained and the resin washed with DMF until the wash solution was colourless, DCM (3x5 mL), MeOH (3x5 mL) and finally ether (3x5 mL).

Cleavage and purification: The resin, pre-swollen in DCM, was treated with a cleavage cocktail of TFA:triisopropylsilane(TIS):water (95:2.5:2.5) for 3h at room temperature. The reaction solution was drained and the resin washed with the cleavage cocktail. The combined solution was precipitated against cold ether, the peptide collected by centrifugation (x3) and purified by RP-HPLC on a C_{18} semi-preparative column. The desired fractions containing the product were collected and lyophilized to afford the products that were characterized by MALDI TOF MS and analytical HPLC.

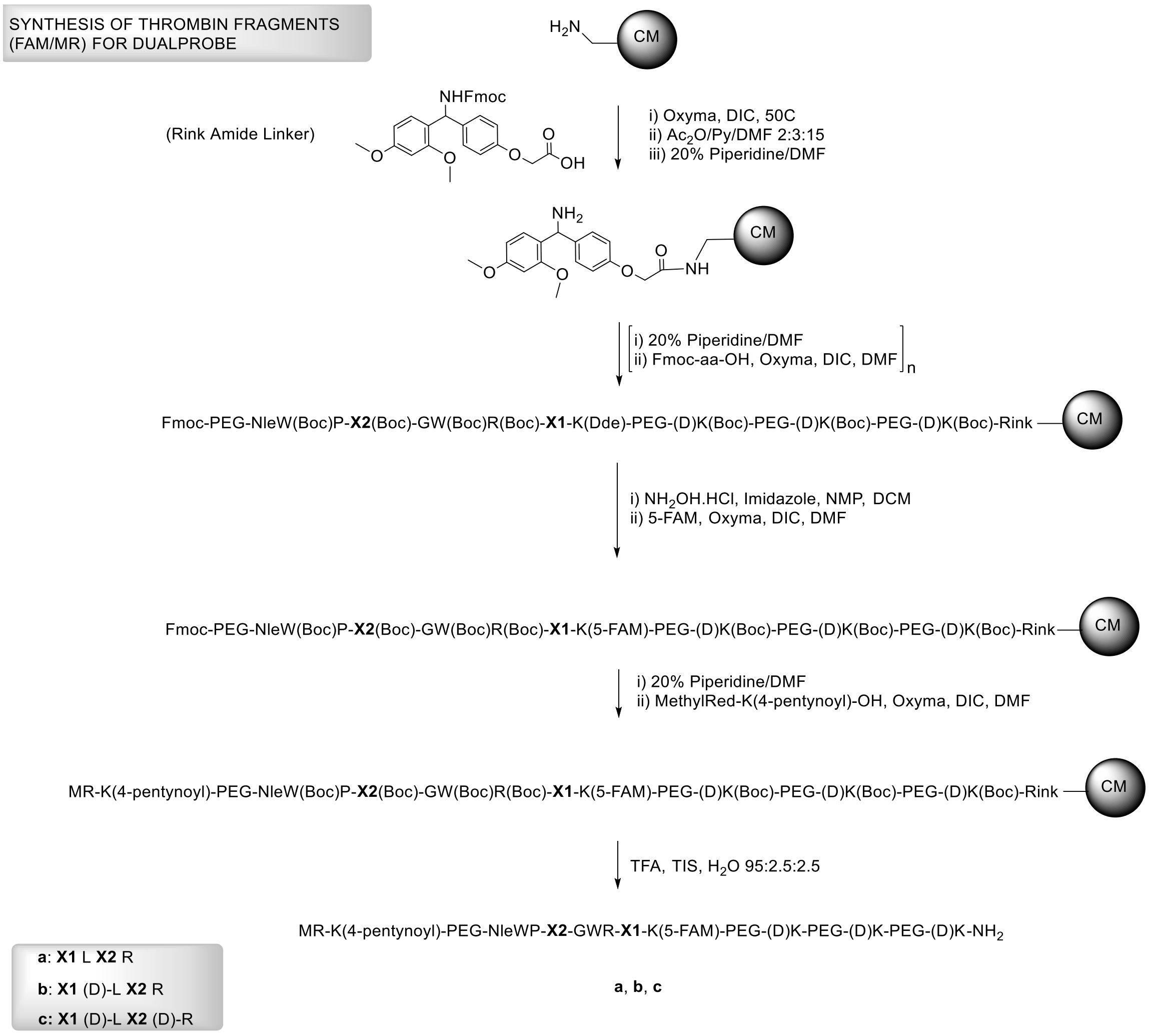

Scheme S2. Synthesis of the FRET Thrombin peptides **a**, **b** and **c** with the FAM, MR and alkyne moieties.

3. Thrombin probes containing the alkyne, FAM & Methyl Red groups. Characterization data (a, b, c)

Thrombin substrates (Scheme S2) were built on the resin, cleaved and purified following the general procedures described above.

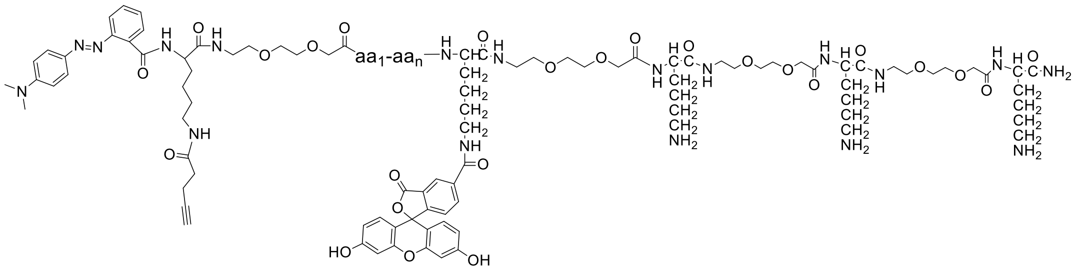

Compound **a**: HPLC t_R = 4.0 min, MALDI calc. for $C_{148}H_{211}N_{34}O_{33}^+$ $[M+H]^+$: 2994.520; found: 2994.846.

Compound **b**: HPLC t_R = 4.2 min, MALDI calc. for $C_{148}H_{211}N_{34}O_{33}^+$ $[M+H]^+$: 2994.520; found: 2994.037.

Compound **c**: HPLC t_R = 4.1 min, MALDI calc. for $C_{148}H_{211}N_{34}O_{33}^+$ $[M+H]^+$: 2994.520; found: 2994.636.

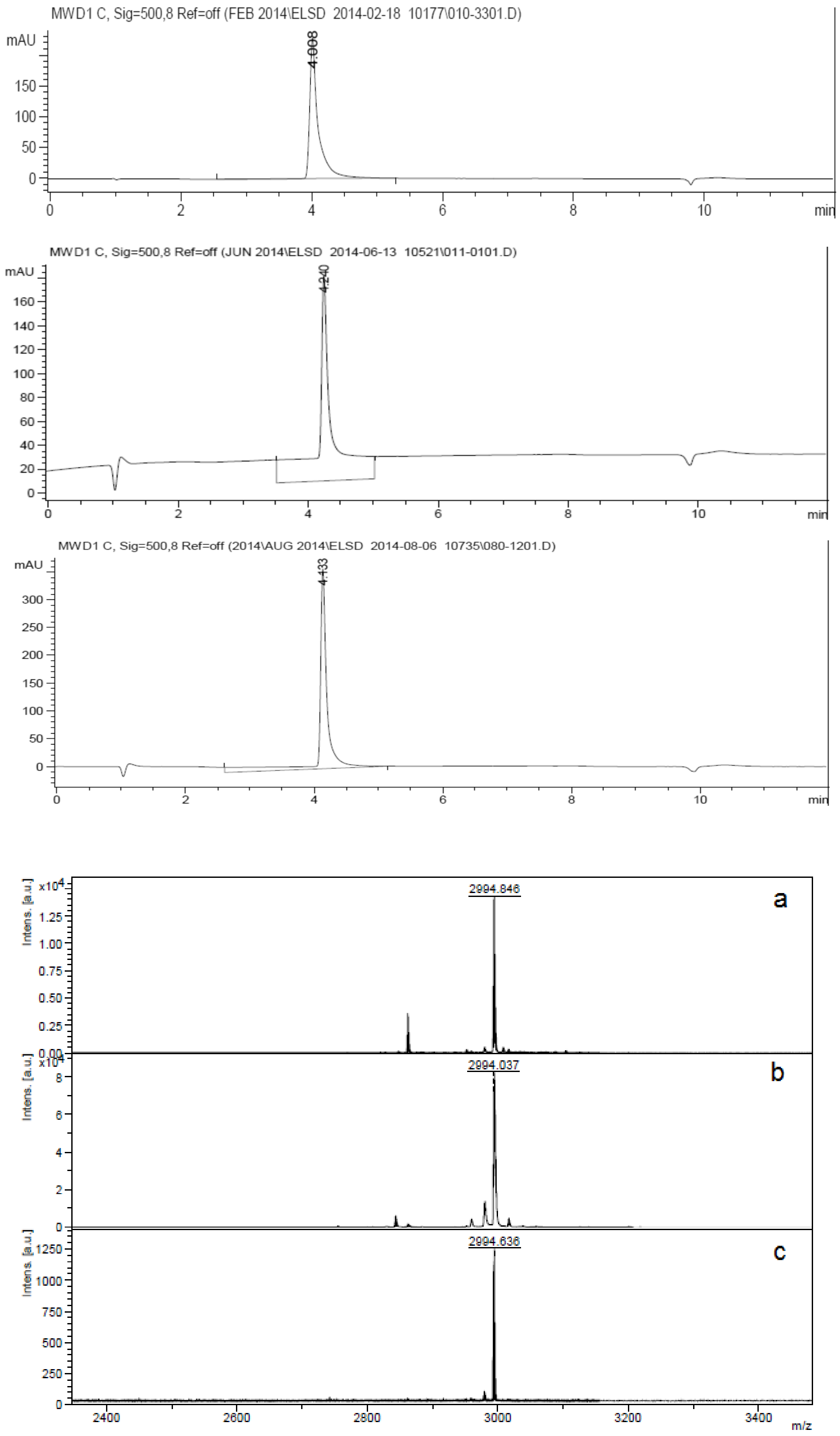

Figure S8. HPLC traces (detection at 500 nm) and MALDI TOF MS analysis for compounds **a** (upper), **b** (middle) and **c** (lower).

4. Synthesis of Methyl Red-Lys-(*N*-4-pentynoyl)-OH

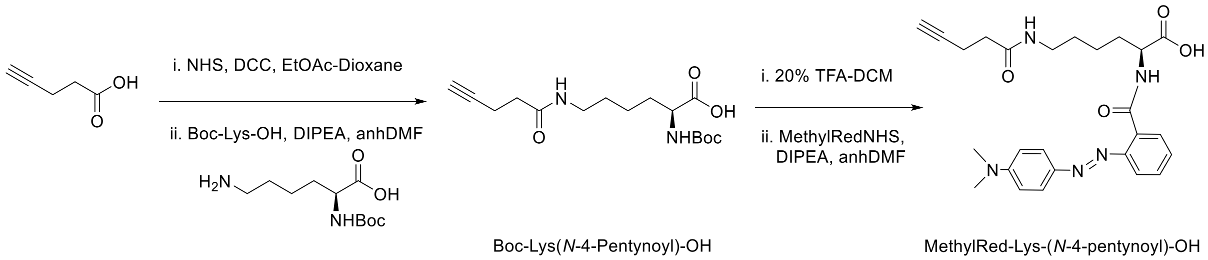

Scheme S3

4-Pentynoic acid succinimidyl ester: A solution of 4-pentynoic acid (0.5 g, 5.1 mmol) and *N*-Hydroxysuccinimide (0.59 g, 1 eq) in EtOAc-Dioxane (1:1, 50 mL) was stirred at 0^0C and DCC (1.0 g, 1 eq) was added allowing the mixture to reach room temperature and kept at these conditions for 12 h. The DCU formed was removed by filtration and the filtrate concentrated under vacuum. EtOAc (100 mL) was added and washed with 5% $NaHCO_3$ (2x40 mL), water (40 mL) and brine (40 mL). After drying over anhydrous Na_2SO_4 and concentrating *in vacuo* the target compound was recrystallized from DCM/Hexane to obtain a white solid that was used in the next step without further purification. **^{1}H-NMR** (400 MHz, $CDCl_3$) δ: 2.90 (t, 2H, *J* 7.0 Hz), 2.86 (s, 4H), 2.64 (td, 2H, *J* 7.0, 2.7 Hz), 2.07 (t, 1H, *J* 2.7 Hz).

Boc-Lys[*N*-4-Pentynoyl]-OH: Boc-Lys-OH (1.25 g, 5.1 mmol) was dissolved in anydrous DMF (15 mL), DIPEA (0.97 mL) was added followed by dropwise addition of a solution of 4-Pentynoic acid succinimidyl ester (5.1 mmol) in anydrous DMF (8 mL). The reaction mixture was stirred for 3h. The solvent was removed under vacuum. To the crude was added HCl 1N (30 mL) and extracted with EtOAc (3x40 mL). The combined organic phases were dried over anhydrous Na_2SO_4 and evaporated under vacuum to afford a white solid (1.36g, 82%). **MS** $(ES)^-$ *m/z* 325 $[M-H]^-$, spectroscopic data identical to those reported previously in the literature.[8]

Methyl Red-Lys-(*N*-4-pentynoyl)-OH: Boc-Lys[*N*-4-Pentynoyl]-OH (1.0 g, 3.0 mmol) was dissolved in 20% TFA in dichloromethane (10 mL) and the resulting mixture was stirred for 3h. The solvent was removed under vacuum and co-evaporated with toluene. The crude was dissolved in anydrous DMF (5 mL). Methyl Red-NHS ester[9] (1.1 g, 1 eq) and DIPEA (1.5 mL, 3eq) were added and the mixture stirred overnight. The solvent was removed under vacuum and the crude mixture dissolved in DCM (150 mL). HCl 1N (100 mL) was added and extracted again with DCM (2x100 mL). The combined organic phases were dried over anhydrous Na_2SO_4, evaporated under vacuum and purified by silica column chromatography (1:10 to 1:3 MeOH/EtOAc) to afford Methyl Red-Lys-(*N*-4-pentynoyl)-OH as a dark red solid (0.67 g, 47%). **m.p.** 153-155^{0}C; **^{1}H-NMR** (500 MHz, CD_3OD) δ: 8.15 (dd, 1H, *J* 7.8, 1.4 Hz), 7.98 (d, 2H, *J* 8.9 Hz), 7.78 (d, 1H, *J* 7.9 Hz), 7.57 (td, 1H, *J* 7.6, 1.3 Hz), 7.49 (t, 1H, *J* 7.6

Hz), 6.86 (d, 2H, *J* 9.1 Hz), 4.65 (t, 1H, *J* 5.2 Hz), 3.12 (s, 6H), 3.08 (t, 2H, *J* 6.1 Hz), 2.41-2.37 (m, 2H), 2.30-2.26 (m, 2H), 2.24 (t, 1H, *J* 2.6 Hz), 2.02 (m, 1H), 1.87 (m, 1H), 1.49 (m, 2H), 1.43(m, 2H); **^{13}C-NMR** (125 MHz, CD_3OD) δ: 179.1, 173.8, 168.2, 155.0, 152.3, 144.8, 133.0, 131.6, 131.0, 130.2, 127.5, 117.4, 112.8, 83.5, 70.4, 56.4, 40.4, 40.3, 36.1, 33.6, 30.1, 24.3, 15.8; **MS** $(ES)^+$ *m/z* 478 $[M+H]^+$; **HPLC** t_R 5.29 min.

5. Synthesis of sulfo-Cy5: 1,3,3-trimethyl-2-((1E,3Z,5E)-3-(5-carboxypyridin-2-yl)-5-(1,3,3-trimethyl-5-sulfonatoindolin-2-ylidene)penta-1,3-dien-1-yl)-3*H*-indol-1-ium-5-sulfonate.

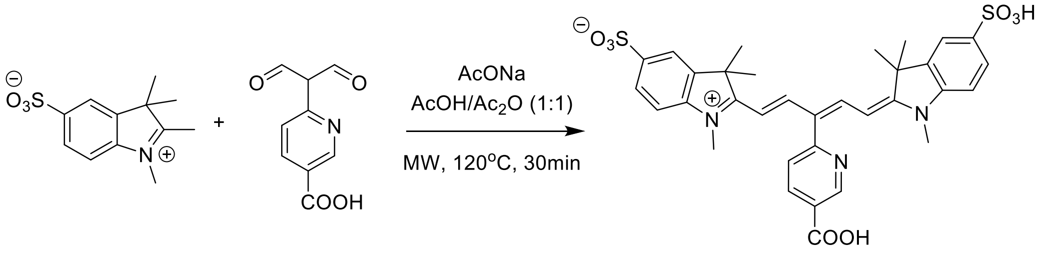

A solution of 1,2,3,3-tetramethyl-3*H*-indolium 5-sulfonate[10] (372 mg, 1.47 mmol, 2.2 eq), 6-(1-formyl-2-oxoethyl)-3-pyridinecarboxylic acid (129 mg, 0.67 mmol, 1.0 eq) and sodium acetate (346 mg, 4.22 mmol, 6.3 eq) in acetic anhydride/acetic acid (1:1, 10 mL) was added to a microwave vial and heated at 120°C for 30 minutes. The mixture was cooled to room temperature. The solvents were removed under vacuum. Cold diethyl ether was added and the solid collected by centrifugation (3x15mL). The obtained solid was dried under vacuum; ^{1}H NMR (500 MHz, DMSO-d_6) δ 9.19 (s, 1H), 8.44 (d, *J* = 14.3 Hz, 2H), 8.31 (d, *J* = 7.8 Hz, 1H), 7.83 (s, 2H), 7.64 (d, *J* = 8.2 Hz, 2H), 7.42 (d, *J* = 7.8 Hz, 1H), 7.30 (d, *J* = 8.3 Hz, 2H), 5.83 (d, *J* = 14.3 Hz, 2H), 3.35 (s, 6H), 1.77 (s, 12H); ^{13}C NMR (125 MHz, CD_3OD) δ 172.1, 157.1, 154.7, 152.2, 145.4, 143.8, 142.6, 139.8, 134.1, 128.0, 126.8, 121.2, 111.7, 102.7, 50.8, 31.7, 27.5; HR-MS (ESI): cal. $C_{33}H_{32}O_8N_3S_2^-$ 662.1636; found: 662.1651 $(M)^-$

6. MMP probes containing the azide, sulfo-Cy5 & QSY21 groups. Synthesis and Characterization data

QSY21-K($\mathbf{X_0}$)-PEG-$\mathbf{X_1}$-$\mathbf{X_n}$-K(sulfo-Cy5)-PEG-(D)K-PEG-(D)K-PEG-(D)K-NH_2

MMP substrates were built on the resin following the general procedure described above. Sulfo-Cy5 and QSY21 labelling was carried out as follows:

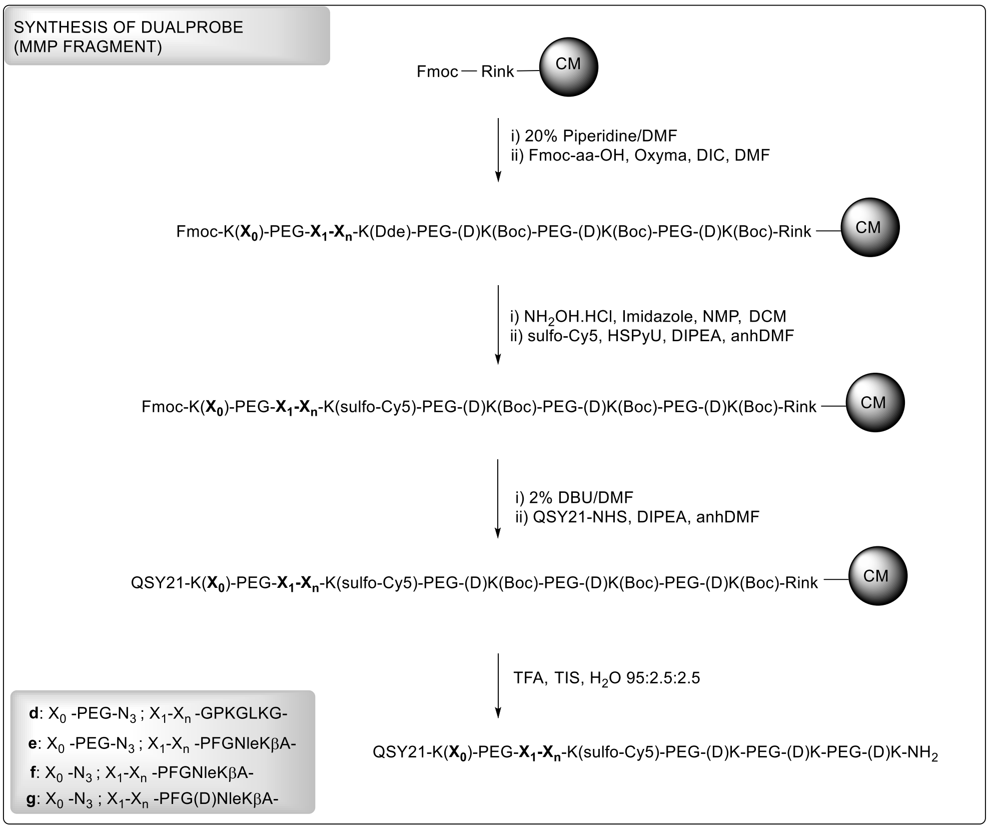

Scheme S4. Synthesis of FRET peptides **d, e, f, g** with sulfo-Cy5 and QSY21 attachment.

The protected peptide sequence built on the Chemmatrix resin [Fmoc-K(X_0)-PEG-$\mathbf{X_1}$-$\mathbf{X_n}$-K(Dde)-[PEG-(D)K(Boc)]$_3$-Rink-Resin] was selectively Dde deprotected according to the general procedure. A solution containing sulfo-Cy5 (1 eq) in anhydrous DMF (10 mg/mL) was activated with N,N,N',N'-bis(tetramethylene)-O-(N-succinimidyl)uronium hexafluorophosphate (HSPyU) (1 eq) and DIPEA (3 eq) at 50^{0}C for 1h. Once the activation was complete the solution was added to the resin together with DIPEA (3 eq) and shaken overnight. The solution was drained and the resin washed with DMF until colourless wash solution, DCM (3x5 mL) and MeOH (3x5 mL). N-terminal Fmoc deprotection was carried out using 2% DBU in DMF (2 x 10 min). In the last step to introduce the quencher, N-terminal capping with the QSY21-NHS ester (1 eq) was carried out in anydrous DMF containing DIPEA (3 eq) for 12 h. The solution was drained and the resin washed with DMF until the wash solution was colourless, DCM (3x5 mL), MeOH (3x5 mL) and finally ether (3x5 mL). Cleavage and purification were done according to the general procedure to obtain the following compounds:

d: HPLC t_R = 3.9 min, MALDI calc. for. $C_{163}H_{232}N_{33}O_{38}S_3^+$ $[M]^+$: 3358.021; found: 3358.851.

e: HPLC t_R = 4.0 min, MALDI calc. for $C_{165}H_{228}N_{31}O_{37}S_3^+$ $[M]^+$: 3333.998; found: 3333.765.

f: HPLC t_R = 4.5 min, MALDI calc. for $C_{159}H_{217}N_{30}O_{34}S_3^+$ $[M]^+$: 3188.840; found: 3188.966.

g: HPLC t_R = 4.4 min, MALDI calc. for $C_{159}H_{217}N_{30}O_{34}S_3^+$ $[M]^+$: 3188.840; found: 3188.904.

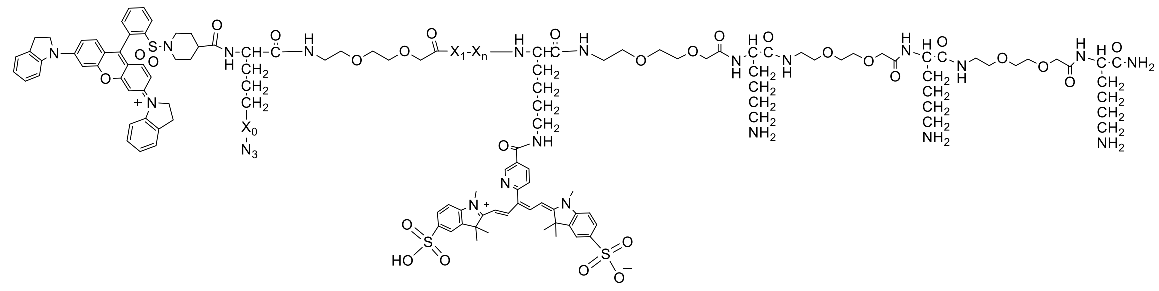

for **d**: X_1-X_n-: -GPKGLKG- ; X_0: $-CH_2-NH-CO-CH_2OCH_2CH_2OCH_2CH_2-$
for **e**: X_1-X_n-:-PFGNleKβA; X_0: $-CH_2-NH-CO-CH_2OCH_2CH_2OCH_2CH_2-$
for **f**: X_1-X_n-: -PFGNleKβA; X_0: $-CH_2-$
for **g**: X_1-X_n-: -PFG(D)NleKβA; X_0: $-CH_2-$

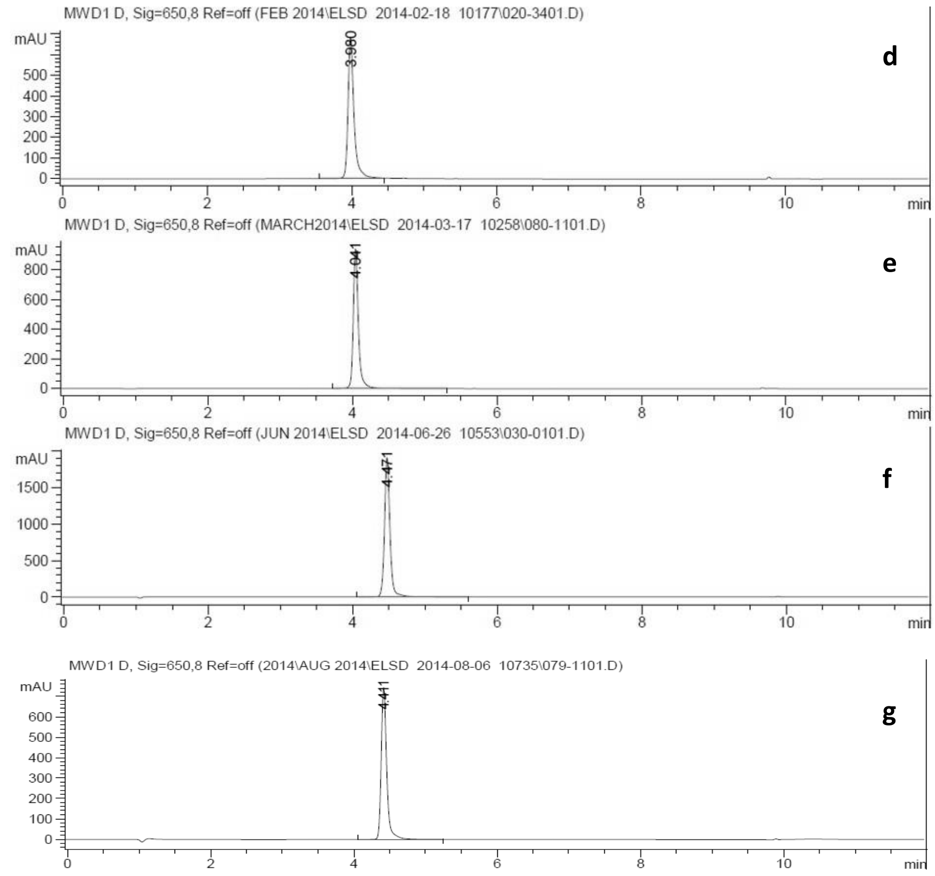

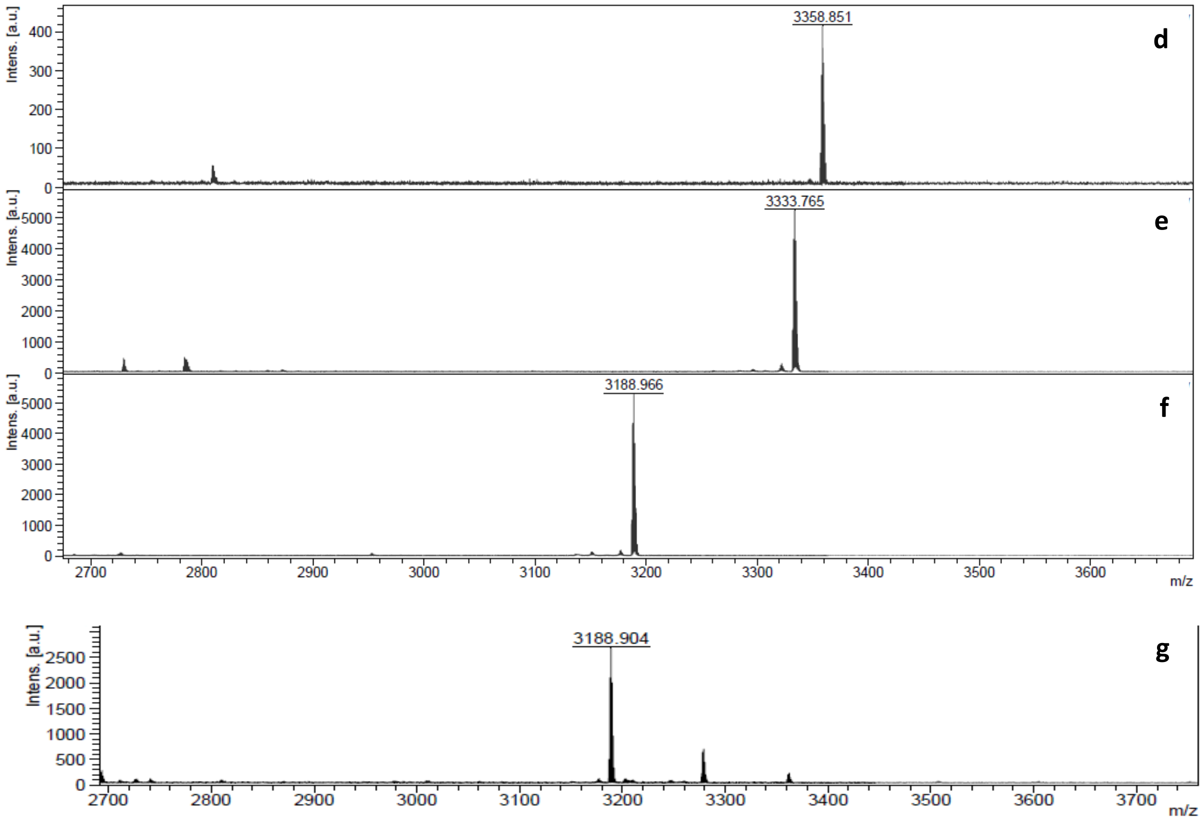

Figure S9. HPLC traces and MALDI TOF MS spectra for compounds **d**, **e**, **f**, **g**.

7. Dual-probes. Synthesis and Characterization data

Table S1

Compound	Thrombin fragment	MMP fragment
**1**	**a**	**d**
**2**	**a**	**e**
**3**	**b**	**f**
**4***	**c**	**f**
**5***	**b**	**g**

* Control probes

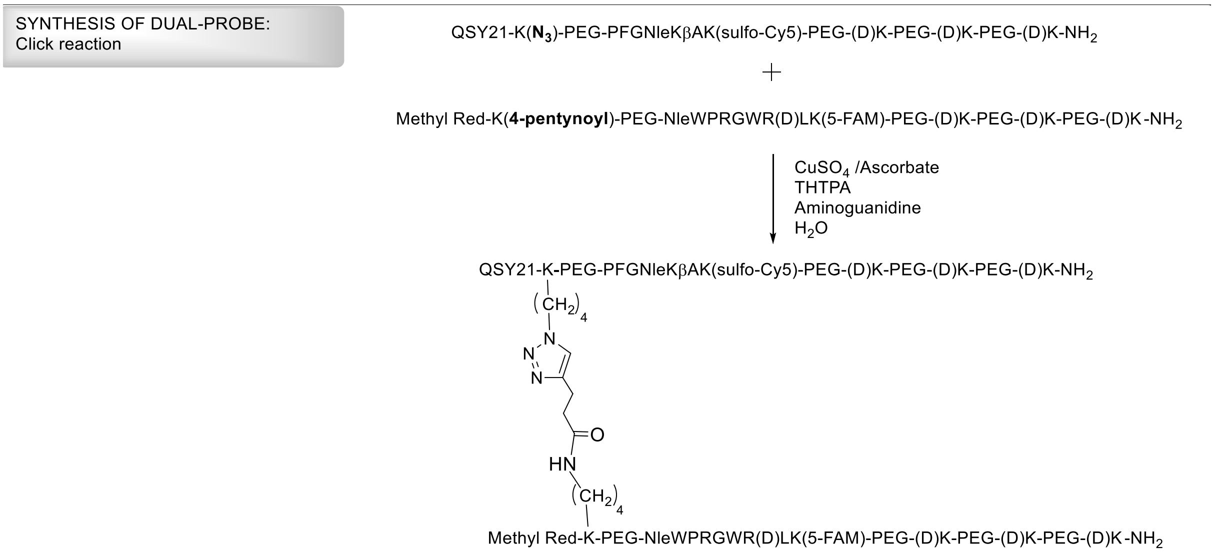

Scheme S5. Synthesis of dual-probes exemplified for compound **3**

General procedure for the fabrication of the dual-probes by Cu-catalysed azide-alkyne cycloaddition chemistry. Optimized conditions for the click reaction were used.[11] In an eppendorf tube the following aqueous reagents were mixed: alkyne-peptide fragment (**a**, **b** or **c**) (50 µL, 1mM), azide-peptide fragment (**d**, **e**, **f** or **g**) (50 µL, 1mM), premixed $CuSO_4$ and THPTA (40 µL $CuSO_4$ 20mM and 80 µL THTPA 50 mM), aminoguanidine hydrochloride (250 µL, 100 mM) and finally sodium ascorbate (250 µL, 100 mM). The reaction was allowed to proceed at 30^{0}C for 5h, the reaction mixture was lyophilised and purified by HPLC to give the final dual-probes, which were characterized by MALDI and analytical HPLC:

1: HPLC t_R = 4.21 min, MALDI calc. for $C_{311}H_{442}N_{67}O_{71}S_3^+$ [M^+]: 6351.534; found: 6351.492.

2: HPLC t_R = 4.24 min, MALDI calc. for $C_{313}H_{438}N_{65}O_{70}S_3^+$ [M^+]: 6327.511; found: 6327.699.

3: HPLC t_R = 4.15 min, MALDI calc. for $C_{307}H_{427}N_{64}O_{67}S_3^+$ [M^+]: 6182.353; found: 6182.343.

4: HPLC t_R = 5.2 min, MALDI calc. for $C_{307}H_{427}N_{64}O_{67}S_3^+$ [M^+]: 6182.353; found: 6182.489.

5: HPLC t_R = 5.3 min, MALDI calc. for $C_{307}H_{427}N_{64}O_{67}S_3^+$ [M^+]: 6182.353; found: 6182.260.

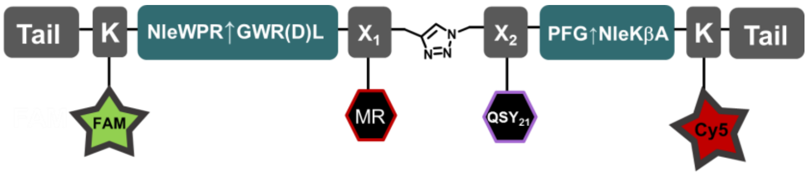

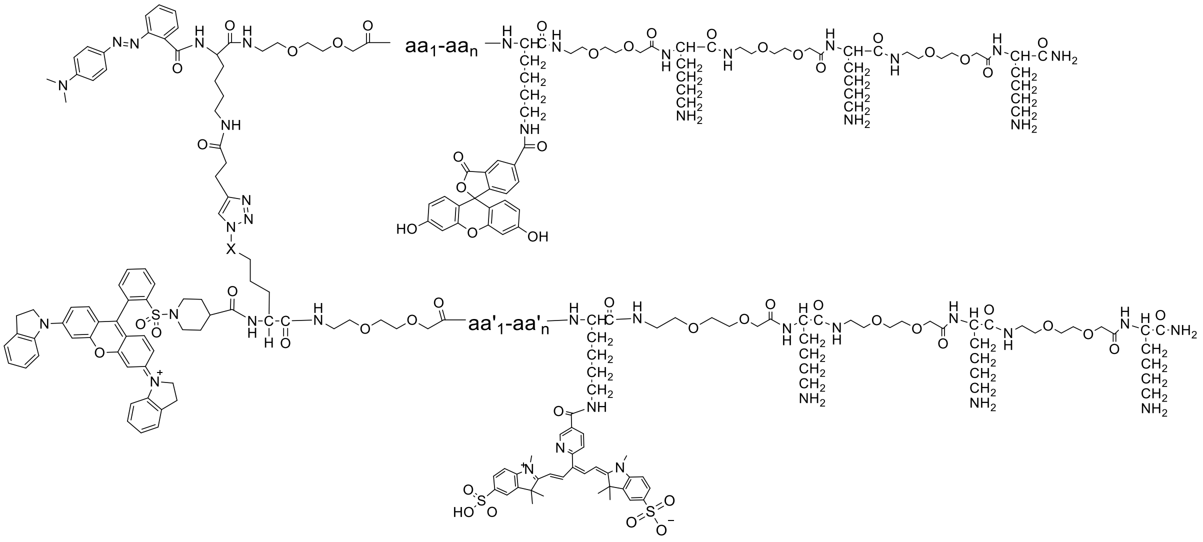

for **1**: -aa$_1$-aa$_n$-: -NleWPRGWRL-; -aa'$_1$aa'$_n$-: -GPKGLKG-; X: -CH_2-NH-CO-$CH_2OCH_2CH_2OCH_2CH_2$-
for **2**: -aa$_1$-aa$_n$-: -NleWPRGWRL-; -aa'$_1$aa'$_n$-: -PFGNleKβA- ; X: -CH_2-NH-CO-$CH_2OCH_2CH_2OCH_2CH_2$-
for **3**: -aa$_1$-aa$_n$-: -NleWPRGWR(D)L-; -aa'$_1$aa'$_n$-: -PFGNleKβA- ; X: -CH_2-
for **4**: -aa$_1$-aa$_n$-: -NleWP(D)RGWR(D)L-; -aa'$_1$aa'$_n$-: -PFGNleKβA- ; X: -CH_2-
for **5**: -aa$_1$-aa$_n$-: -NleWPRGWR(D)L-; -aa'$_1$aa'$_n$-: -PFG(D)NleKβA- ; X: -CH_2-

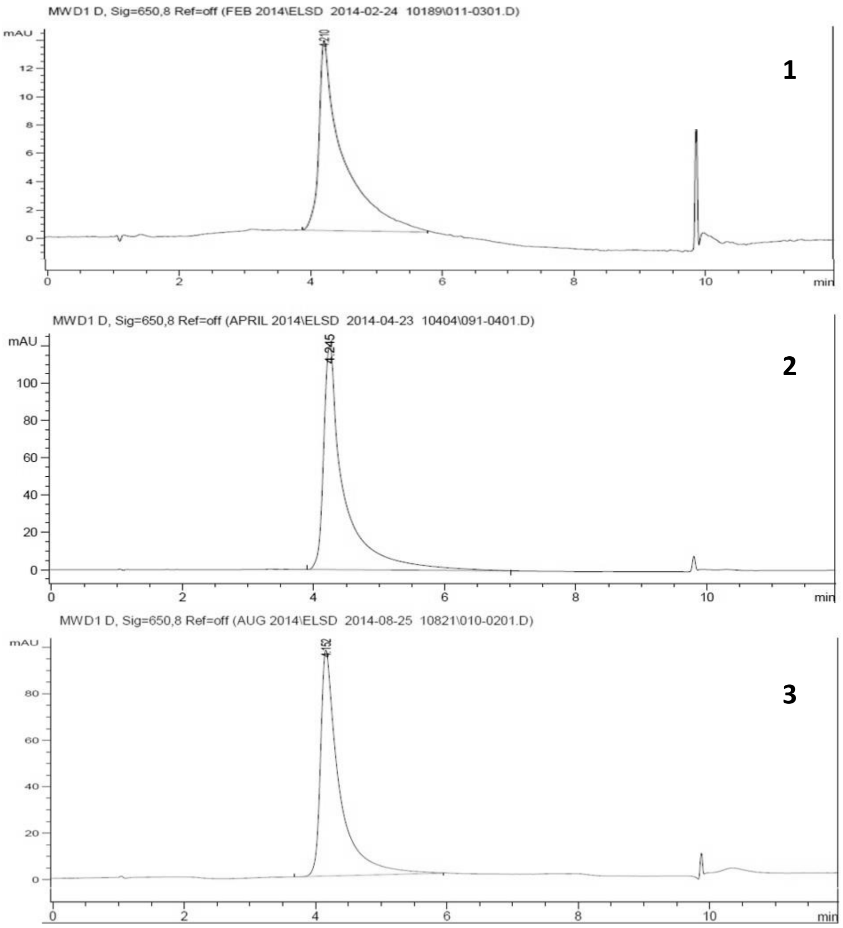

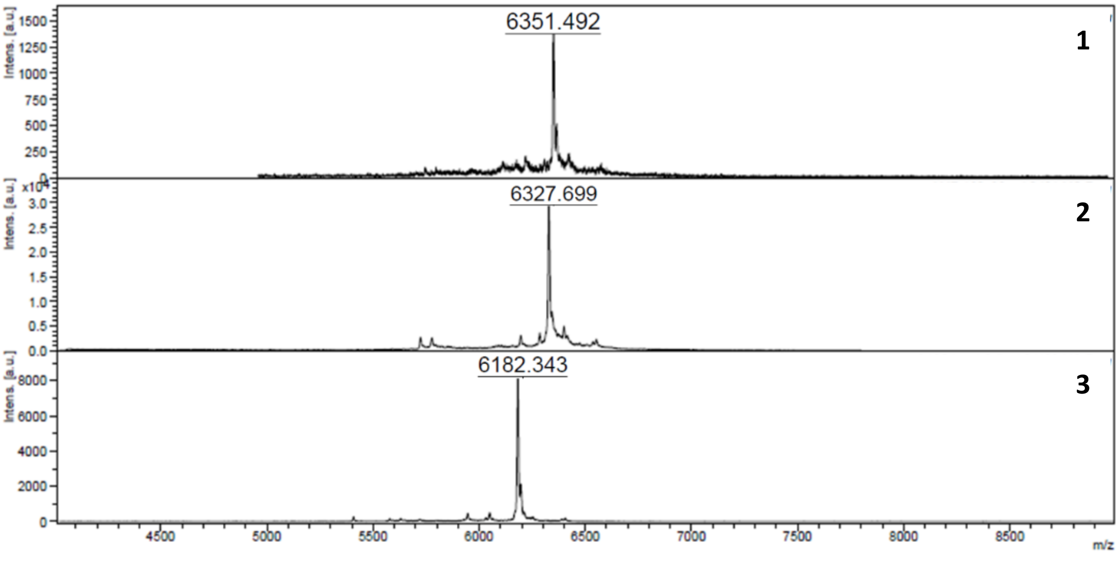

Figure S10. Structure, HPLC traces and MALDI TOF MS spectra for dual-probes **1-3**

Control compounds 4 and 5:

Control compounds were synthesised using D-Arg in the Thrombin cleavage site for **4** or D-Nle in the MMP cleavage site for **5**

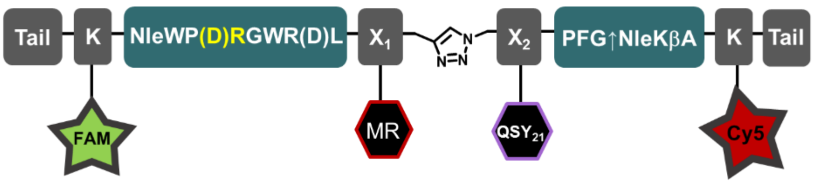

4

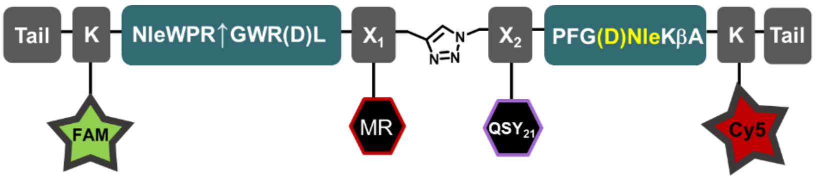

5

Figure S11. Structure of Control probes **4** and **5**

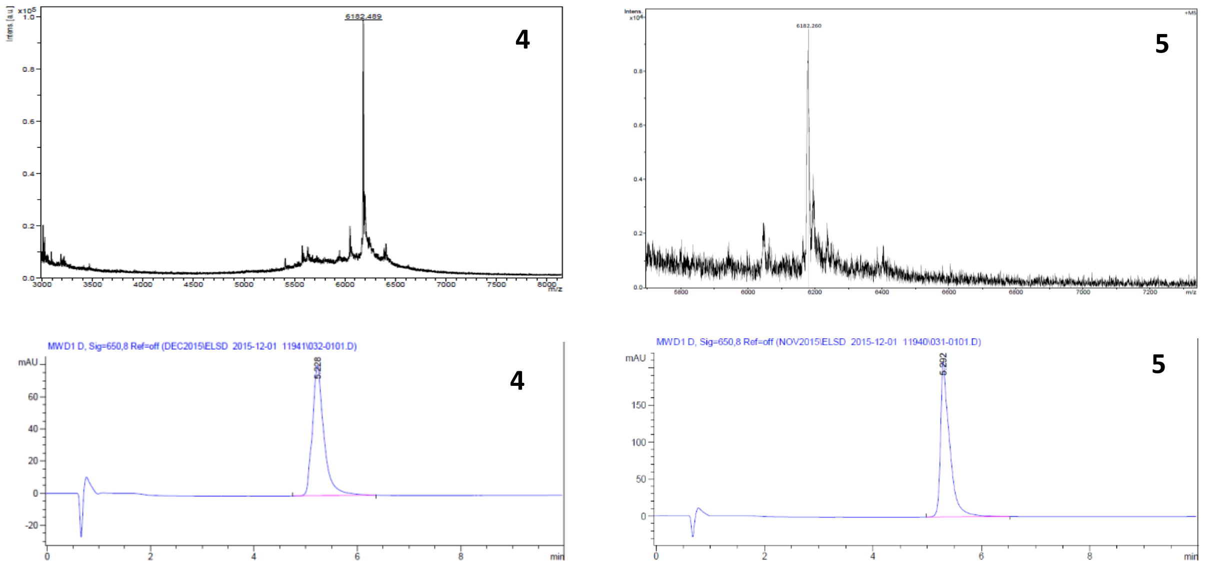

Figure S12. HPLC traces and MALDI TOF MS spectra for compounds **4** and **5**

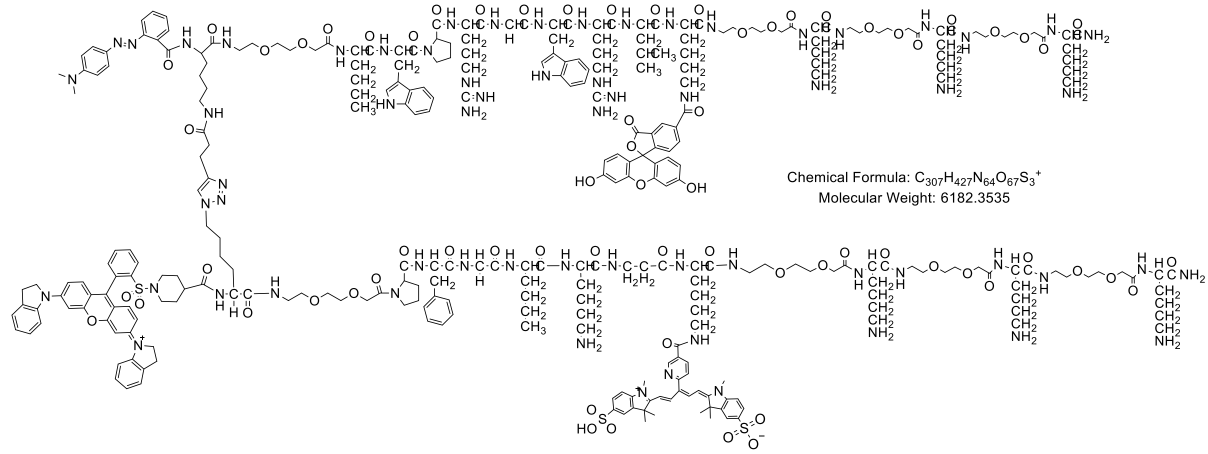

Chemical Formula: $C_{307}H_{427}N_{64}O_{67}S_3^+$
Molecular Weight: 6182.3535

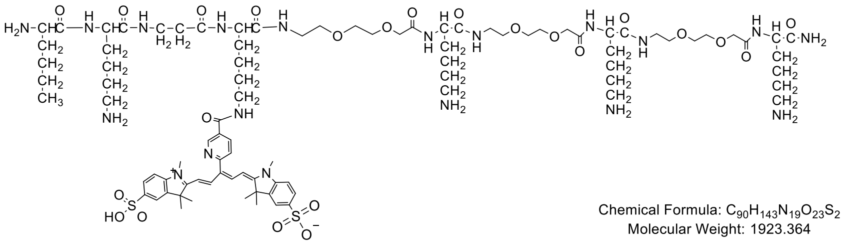

Chemical Formula: $C_{90}H_{143}N_{19}O_{23}S_2$
Molecular Weight: 1923.364

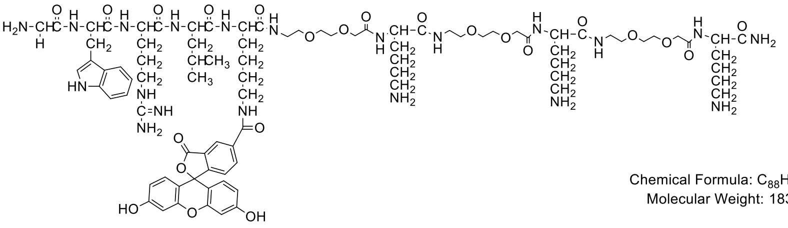

Chemical Formula: $C_{88}H_{130}N_{20}O_{23}$
Molecular Weight: 1836.1250

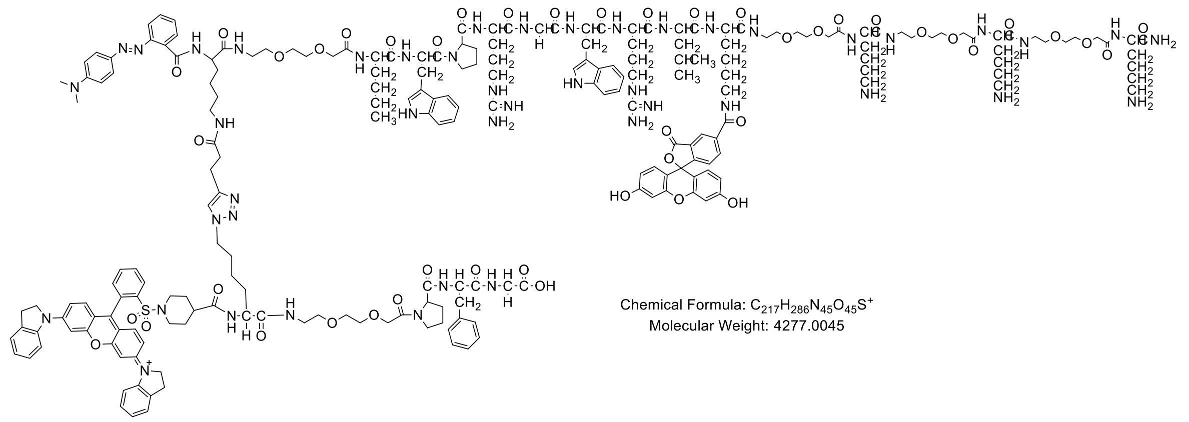

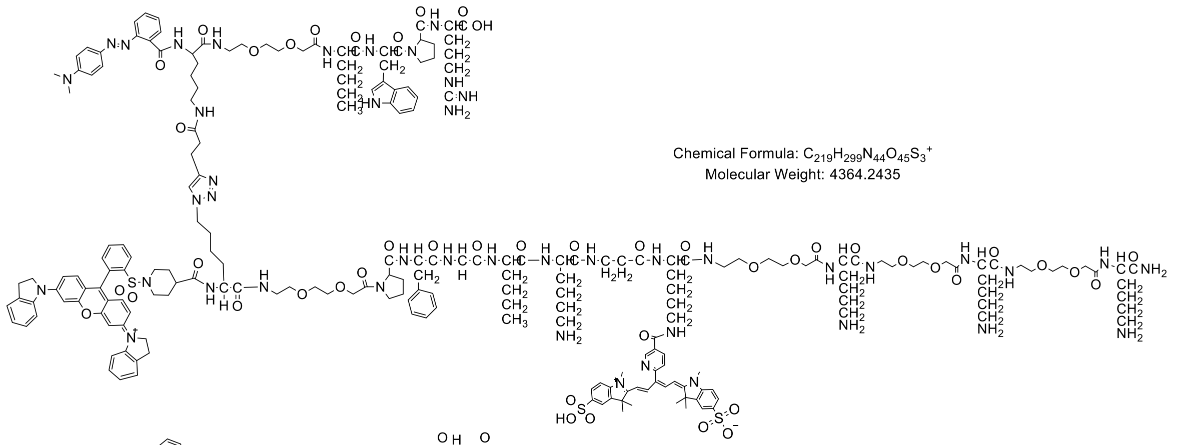

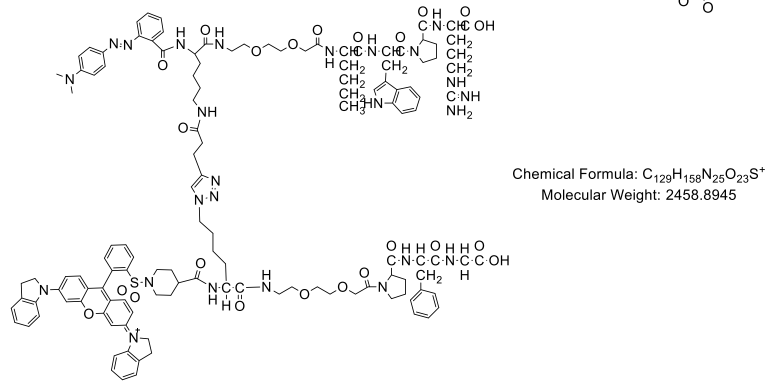

Figure S13. Chemical structures of the fragments detected by MALDI-TOF MS after enzymatic treatment of compound **3**.

8. Characterisation Table

Table S2

	COMPOUND	m/z_{calc} (Da)*	MALDI m/z (Da)	HPLC t_R (min)
**a**	MR-K(Alkyne)-PEG_2-NleWPRGWRL-K(FAM)-(PEG_2-(D)K-$)_3$-NH_2	2994.520	2994.846	4.008
**b**	MR-K(Alkyne)-PEG_2-NleWPRGWR(D)L-K(5-FAM)-(PEG_2-(D)K-$)_3$-NH_2	2994.520	2994.037	4.240
**c**	MR-K(Alkyne)-PEG_2-NleWP(D)RGWR(D)L-K(5-FAM)-(PEG_2-(D)K-$)_3$-NH_2	2994.520	2994.636	4.133
**d**	QSY21-K(PEG_2-**N_3**)-PEG_2-GPKGLKG-K(Cy5)-(PEG_2-(D)K-$)_3$-NH_2	3358.021	3358.851	3.980
**e**	QSY21-K(PEG-**N_3**)-PEG_2-PFGNleKβA-K(Cy5)-(PEG_2-(D)K-$)_3$-NH_2	3333.998	3333.765	4.041
**f**	QSY21-K(**N_3**)-PEG_2-PFGNleKβA-K(Cy5)-(PEG_2-(D)K-$)_3$-NH_2	3188.840	3188.966	4.471
**g**	QSY21-K(**N_3**)-PEG_2-PFG(D)NleKβA-K(Cy5)-(PEG_2-(D)K-$)_3$-NH_2	3188.840	3188.904	4.411
**1**	QSY21-K(PEG)-PEG_2-GPKGLKG-K(Cy_5)-(PEG_2-(D)K-$)_3$-NH_2<br>| triazole<br>| MR-K-PEG_2-NleWPRGWRLK(FAM)-(PEG_2-(D)K-$)_3$-NH_2	6351.534	6351.492	4.210
**2**	QSY21-K(PEG_2)-PEG_2-PFGNleKβA-K(Cy5)-(PEG_2-(D)K-$)_3$-NH_2<br>| triazole<br>| MR-K-PEG_2-NleWPRGWRLK(FAM)-(PEG_2-(D)K-$)_3$-NH_2	6327.511	6327.699	4.245
**3**	QSY21-K-PEG_2-PFGNleKβA-K(Cy5)-(PEG_2-(D)K-$)_3$-NH_2<br>| triazole<br>| MR-K-PEG_2-NleWPRGWR*(D)*LK(5-FAM)-(PEG_2-(D)K-$)_3$-NH_2	6182.353	6182.343	4.152

**4**	QSY21-K-PEG$_2$-PFGNleKβA-K(Cy5)-(PEG$_2$-(D)K-)$_3$-NH$_2$<br>triazole<br>MR-K-PEG$_2$-NleWP(D)RGWR*(D)*LK(5-FAM)-(PEG$_2$-(D)K-)$_3$-NH$_2$	6182.353	6182.489	5.228
**5**	QSY21-K-PEG$_2$-PFG(D)NleKβA-K(Cy5)-(PEG$_2$-(D)K-)$_3$-NH$_2$<br>triazole<br>MR-K-PEG$_2$-NleWPRGWR*(D)*LK(5-FAM)-(PEG$_2$-(D)K-)$_3$-NH$_2$	6182.353	6182.260	5.292

* m/z calculated for $[M+H]^+$ or $[M]^+$

Biological Materials and Methods:

Murine LPS model: Lipopolysaccharide (LPS) lung injury and lavage collection and processing was performed as previously described,[14] however lavage was collected 24h post LPS administration (*Escherichia coli* serotype O111:B4; Sigma-Aldrich). All mice were female 8-10 week old CD1. Three were dosed with LPS, three were naïve controls. Presence of active MMP in the lavage fluid was confirmed by zymography (Novex® 10% Zymogram (Gelatin) Protein Gels, 1.0 mm, 15 well, Thermo Scientific). Gels were run at 150 kV 4 °C, renatured 4 °C (Novex™ Zymogram Renaturing Buffer (10X), Thermo Scientific) 90 min, followed by developing (Novex™ Zymogram Developing Buffer (10X) buffer, Thermo Scientific) overnight at 37 °C (with 50 µM Marimastat when appropriate). Gels were stained with Simply Blue Safe Stain (Thermo Scientific) according to manufacturer's instructions. PRP was harvested from whole murine blood as described above for human PRP.

Plate reader assays were performed as described in the main text, with the MMP buffer replaced with lavage fluid or PRP. All experiments were carried-out in duplicate. Data was normalised by background subtraction of intrinsic fluorescence.

PEPTIDE SEQUENCE OPTIMIZATION OF THE MMP-2/9/13 SUBSTRATE

To generate molecular probe sequences to optimally/specifically measure MMP activity the probes were synthesised as shown below:

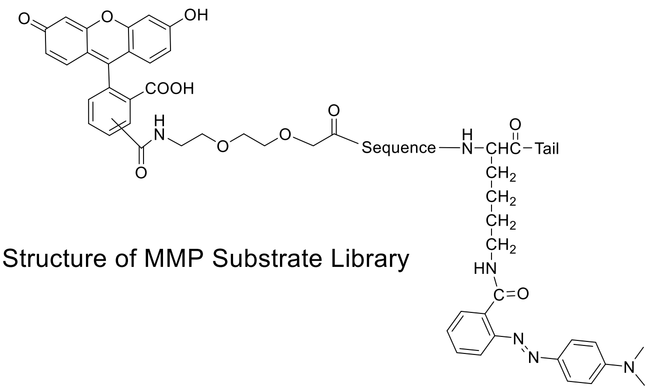

Structure of MMP Substrate Library

Seq ID No.	Generation 1	Notes	Generation 2	Generation 3	Generation 4
	*-NH_2		*-$(PEG_2)_2$-NH_2	*-K-K-$(PEG_2)_2$-NH_2	*-[PEG_2-(D)K$]_3$-NH_2
1	G-P-K-*G*-*L*-K-G	→	1-G2	1-G3	
2	G-P-K-G-(D)L-K-G	Control sequence			
3	G-P-K-*G*-*I*-K-G	Cleaved by elastase			
4	G-P-K-*G*-*Nle*-K-G				
5	P-F-*G*-*M*-K-βA				
6	P-F-G-(D)M-K-βA	Control sequence			
7	P-F-*G*-*L*-K-βA				
8	P-F-*G*-*I*-K-βA	Cleaved by elastase			
9	P-F-*G*-*Nle*-K-βA	→	9-G2	9-G3	9-G4
10	P-Cha-*G*-*M*-F-G				
11	P-Cha-*G*-*M*-W-G				
12	P-Cha-*G*-*M*-Y(Me)-G				
13	P-Cha-*G*-*M*-Y-G				
14	P-Cha-*G*-*M*-K-βA-G				
15	P-Cha-*G*-*M*-H-G				
16	P-Cha-*G*-*M*-K-G				

Table S3. Library of the FRET compounds generated/screened and iterated from the first to fourth generation. The MMP cleavage site is indicated by italics. *Structure of the tail for each generation of probe.

The initial library of FRET activatable probes (Table S3 - Generation 1 probes) contained the fluorophore 5,6-Carboxyfluorescein and the quencher Methyl Red separated by a peptide sequence acting as the substrate for the target enzyme. Sequence 1 was selected according to a previous proteomic study using iTRAQ-TAILS.[15] Other sequences were designed and included in the initial G1 screen after analysis of commercial and other reported MMP substrates.

The FRET peptides were synthesized by manual standard solid-phase Fmoc chemistry and evaluated as MMP substrates. The response towards MMP and the specific inhibition with the MMP inhibitor

Marimastat was measured (Fig S14), with site specific cleavage confirmed for all the sequences (Table S3) by MALDI-TOF MS analysis. The others MMPs tested shared the same cleavage site.

Specific inhibition of fluorescence signal using Marimastat (a pan-MMP inhibitor) was successful for all the probes tested. Control probes (sequence **2** and **6**) containing D-aminoacids in the cleavage site showed no increase in fluorescence and no cleavage was detected by MALDI TOF MS.

Selectivity was determined by analysis of the change in fluorescence over background upon the addition of different proteases (MMP -2, -9, -12, -13, Neutrophil elastase and Thrombin). In order to choose the best probes for *in vivo or ex vivo use* a number of additional parameters were evaluated which included fluorescence increase in the presence of other related inflammatory proteases such as Thrombin and human Neutrophil Elastase (NE) (Fig S15 and Table S4).

Results from the generation 1 experiments indicated that sequences **3** and **8** were cleaved by NE, clearly a major issue for a probe for use with tissue. MALDI TOF MS analysis performed after enzymatic treatment confirmed the probes were being cleaved by NE at a different site to those found for the MMPs. NE cleaved probe **3-G1** at -G-P-K-G-I↑K-G- and probe **8-G1** at P-F-G-I↑K-βA (Fig S16-S17). The other sequences remained intact and Thrombin did not cleave any of the sequences.

The probes containing Methionine in the cleavage site (sequences **5** and **10-16**), which were selectively cleaved by MMPs were deprioritised due to the anticipated stability issues that thioether oxidation can cause. Assays with with human tissue homogenate confirmed specific cleavage and inhibition with Marimastat only for sequence **1** (**-G-P-K-G↑L-K-G-**) and sequence **9** (**-P-F-G↑Nle-K-ßA-**) (Fig S20-S21). All these assays were conducted using standard FRET peptides prior to lead optimisation for incorporation into the dual-probes.

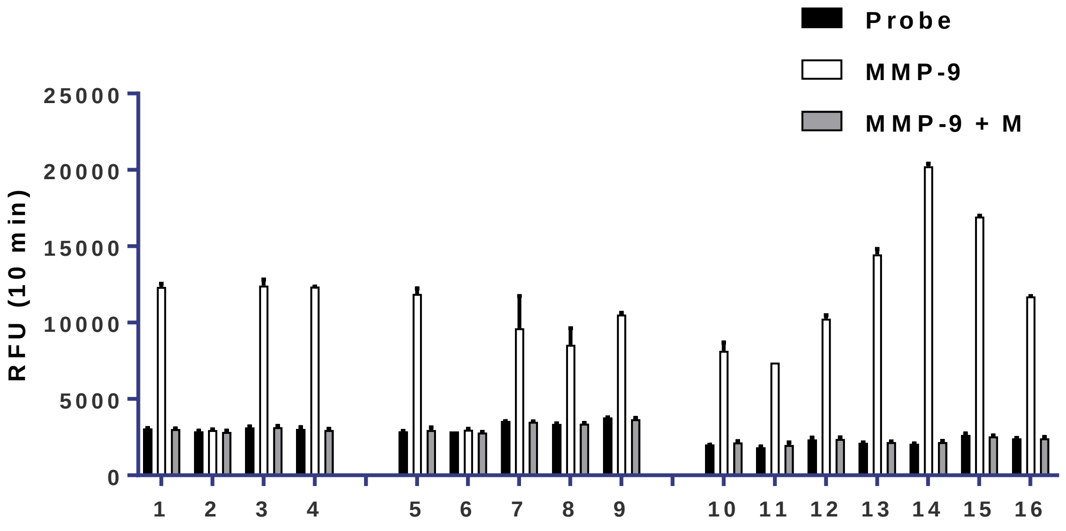

Figure S14. Probe fluorescence (ex 485nm/em 528nm) was measured for all Generation 1 compounds (10µM) with MMP-9 (30nM) in the absence or presence of Marimastat (M) (20µM).

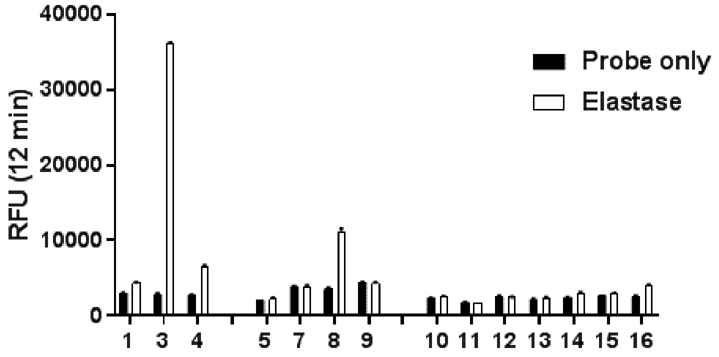

Figure S15. Probe fluorescence (ex 485nm/em 528nm) as measured after 12 min for the Generation 1 compounds (10µM) in the absence or presence of Elastase (30nM).

		Inflammatory mediators						
SEQ ID NO.	Probe sequence	MMP-9 (39.0kDa)	MMP-2 (20.3kDa)	MMP-12 (20.3kDa)	MMP-13 (20.4kDa)	Neutrophil lysate	Neutrophil elastase	Thrombin
**1**	**-G-P-K-*G*-*L*-K-G-**	**3.50**	**1.98**	**2.34**	**9.30**	**1.93**	**1.30**	**1.31**
**3**	-G-P-K-*G*-***Ile***-K-G-	4.22	2.10	1.54	9.33	13.49	11.97	1.37
**4**	-G-P-K-*G*-***Nle***-K-G-	4.53	2.70	1.46	11.75	3.06	1.97	1.48
**5**	-P-F-*G*-***M***-K-ßA-	5.21	3.11	1.88	13.57	1.07	1.06	0.99
**7**	-P-F-*G*-***L***-K-ßA-	2.50	1.87	3.03	6.41	0.99	0.10	0.89
**8**	-P-F-*G*-***Ile***-K-ßA-	2.29	1.59	1.65	5.05	3.29	2.25	0.96
**9**	**-P-F-*G*-*Nle*-K-ßA-**	**2.02**	**1.88**	**1.53**	**5.27**	**0.96**	**0.99**	**0.96**
**10**	-P-Cha-*G*-*M*-**F**-G-	5.89	3.27	2.45	11.75	2.02	1.07	0.94
**11**	-P-Cha-*G*-*M*-**W**-G-	5.07	2.59	2.59	6.5	0.84	1.04	1.05
**12**	-P-Cha-*G*-*M*-**Y(Me)**-G-	4.94	2.54	2.15	10.04	0.75	1.00	0.97
**13**	-P-Cha-*G*-*M*-**Y**-G-	6.90	4.07	2.87	11.26	1.89	1.08	1.04
**14**	-P-Cha-*G*-*M*-**K-ßA**-G-	9.61	5.22	3.21	15.9	1.40	1.19	1.00
**15**	-P-Cha-*G*-*M*-**H**-G-	7.53	4.01	3.05	12.93	1.27	1.08	0.97
**16**	-P-Cha-G-M-**K**-G-	8.21	4.24	2.61	14.88	1.95	1.44	1.02

Table S4. Relative enzymatic specificity of the Generation 1 compounds (at 10 µM) with excitation/emission at 485/528nm. Recombinant human catalytic domain MMPs -2, -9, -12, -13 were used at 30nM. Neutrophil elastase was used at 30 nM, neutrophil lysate was used neat. Recombinant human Thrombin was used at 5U/ml. Cleavage site for each sequence is indicated in italics. As shown below probe 1 was rapidly cleaved by Plasmin while 9 was observed to be stable to Plasmin.

Seq ID No. **3** (G-P-K-G-I-K-G)
+
Elastase

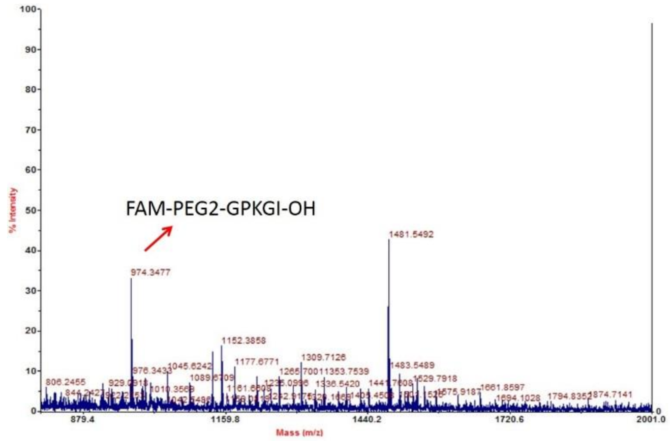

Figure S16. Seq ID No. 3 was cleaved with elastase (G-P-K-G-I ↑ K-G)

Seq ID No. **8** (P-F-G-I-K-βA)
+
Elastase

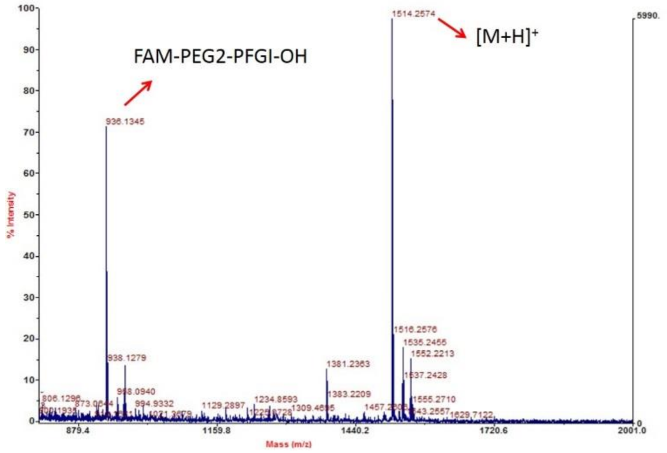

Figure S17. Seq ID No. 8 was cleaved with elastase (P-F-G-I ↑ K-ßA)

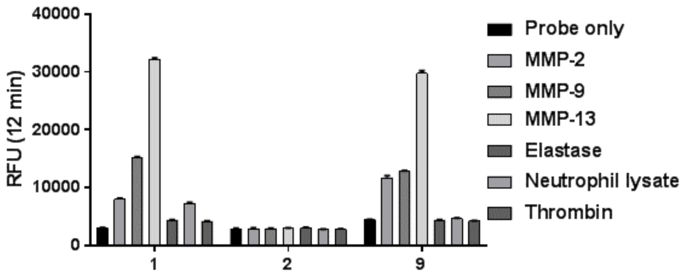

Figure S18. Seq ID No. 1 and 9 were cleaved by MMP-2, -9 and -13 but not with Elastase, Neutrophil lysate and Thrombin, while control compound **2** was not cleaved with any of these proteases as expected.

Seq ID No. **1** (GPKGLKG)

Seq ID No. **1** (GPKGLKG)
+
MMP-9

Seq ID No. **1** (GPKGLKG)
+
Elastase

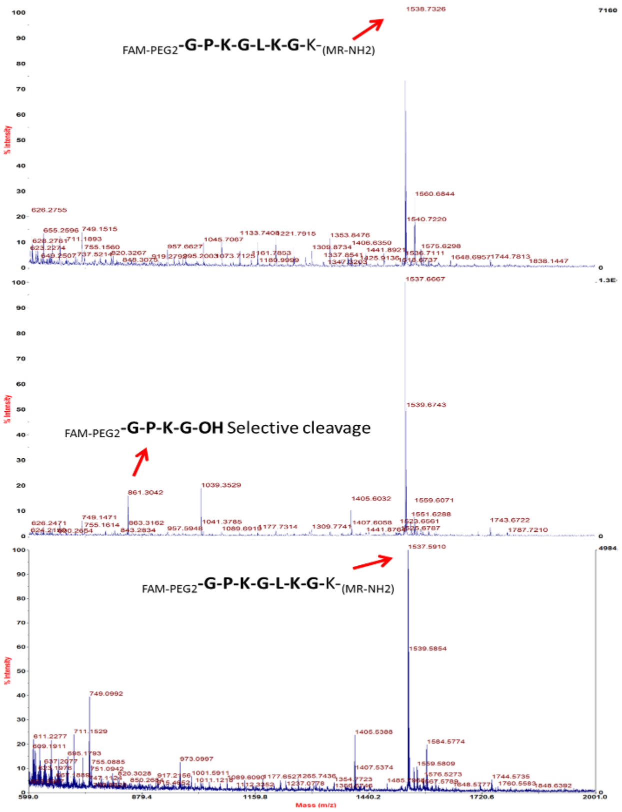

Seq ID No. **9** (PFGNleKβA)

Seq ID No. **9** (PFGNleKβA)
+
MMP-9

Seq ID No. **9** (PFGNleKβA)
+
Elastase

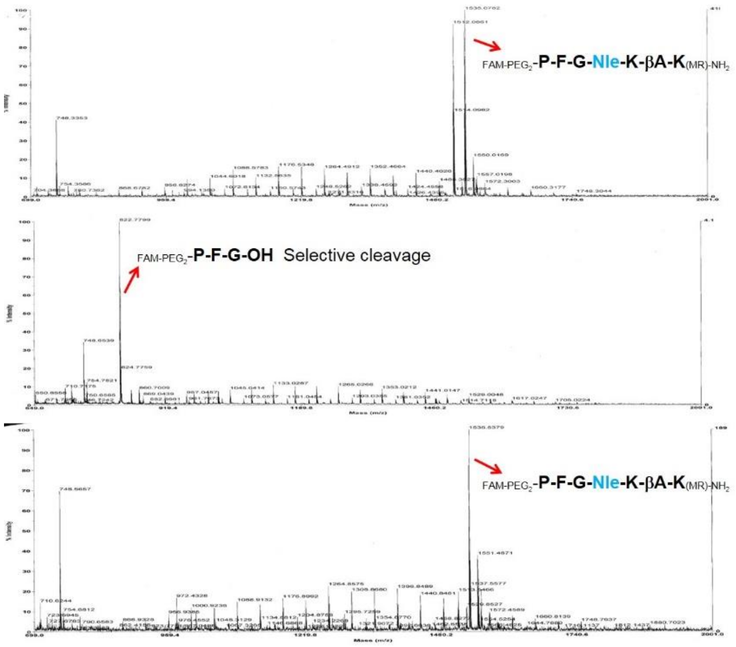

Figure S19. Seq ID No. 1 (GPKGLKG) and No. 9 (PFGNleKβA) were cleaved by MMP-9 and stable to Elastase as showed by MALDI TOF MS analysis.

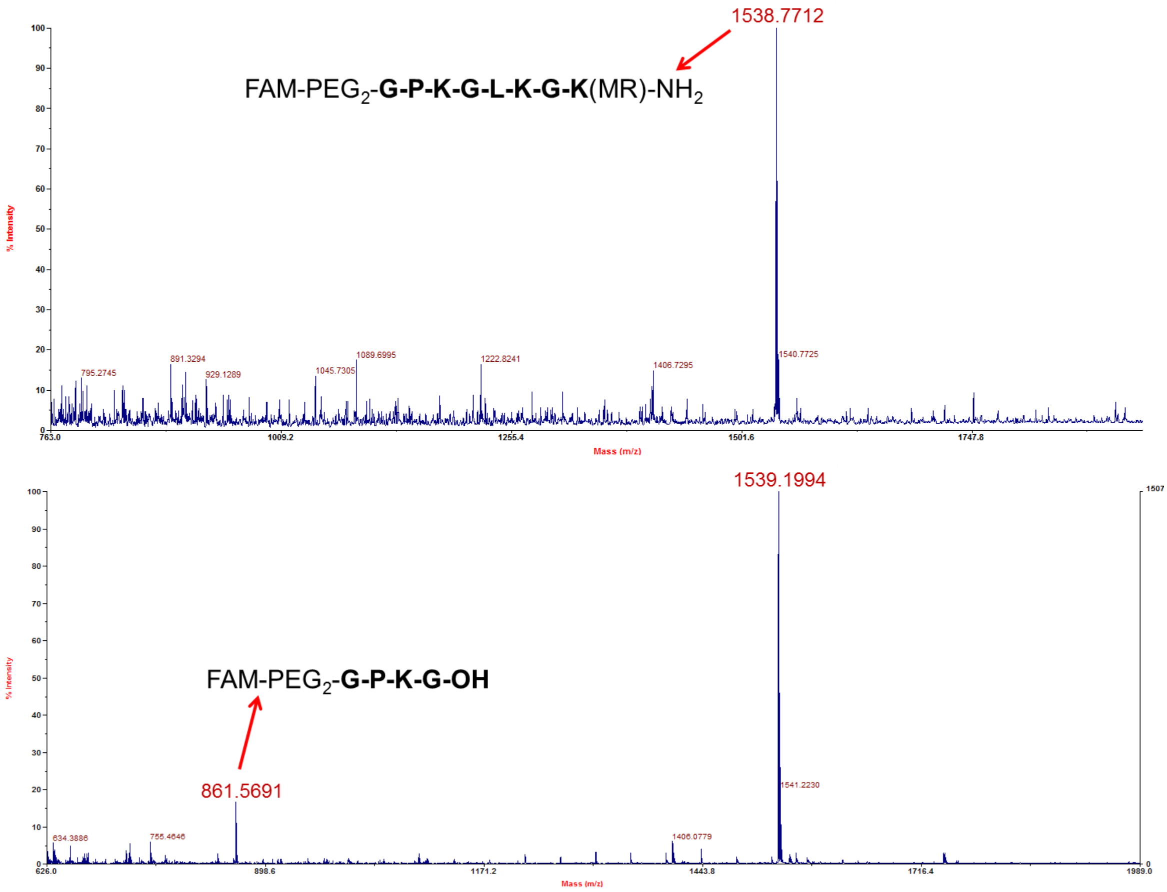

Figure S20. MALDI TOF MS of compound 1-G1 with healthy (upper) and fibrotic lung tissue (lower)

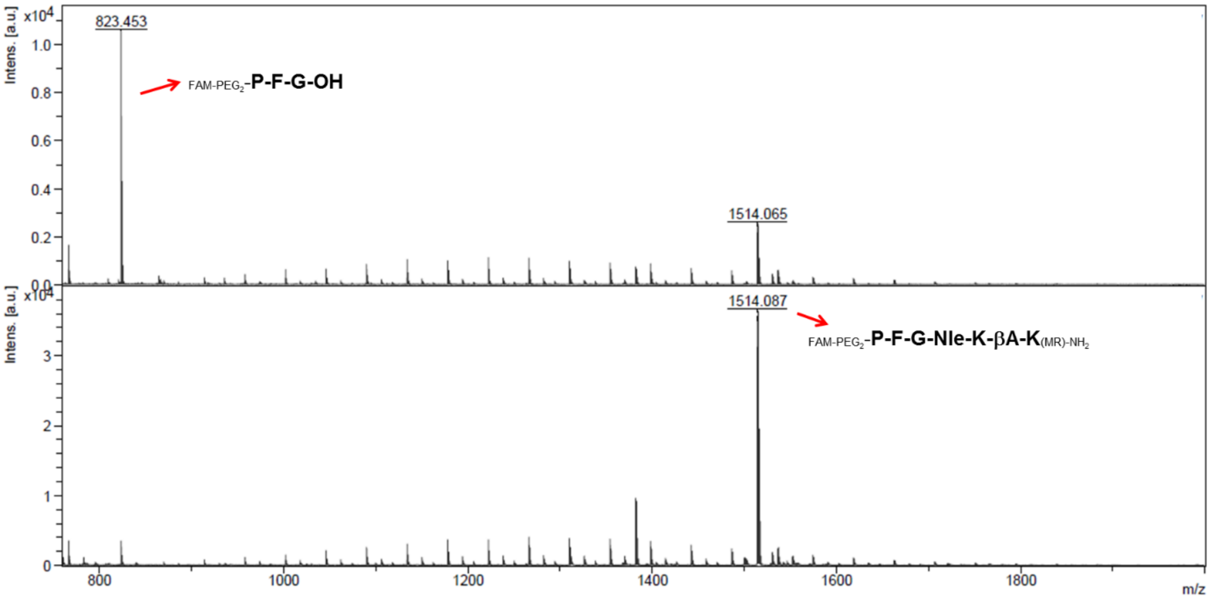

Figure S21. MALDI TOF MS of compound 9-G1 with fibrotic lung tissue (upper) and tissue incubated with Marimastat (lower)

<u>Next Generation Probes:</u>

For the next generation probes sequences 1 and 9 were incorporated into peptides that were stabilised with various hydrophilic tails (added in order to improve the aqueous solubility and prevent exopeptidase cleavage. The two selected sequences 1 and 9 were thus flanked by ethylenglycol units (8-amino-3,6-dioxaoctanoic acids) and Lys or D-Lys residues giving the Generation-2, -3 and -4 compounds (Figure S22).

With the second generation of probes, the selectivity for MMPs and Plasmin was evaluated.

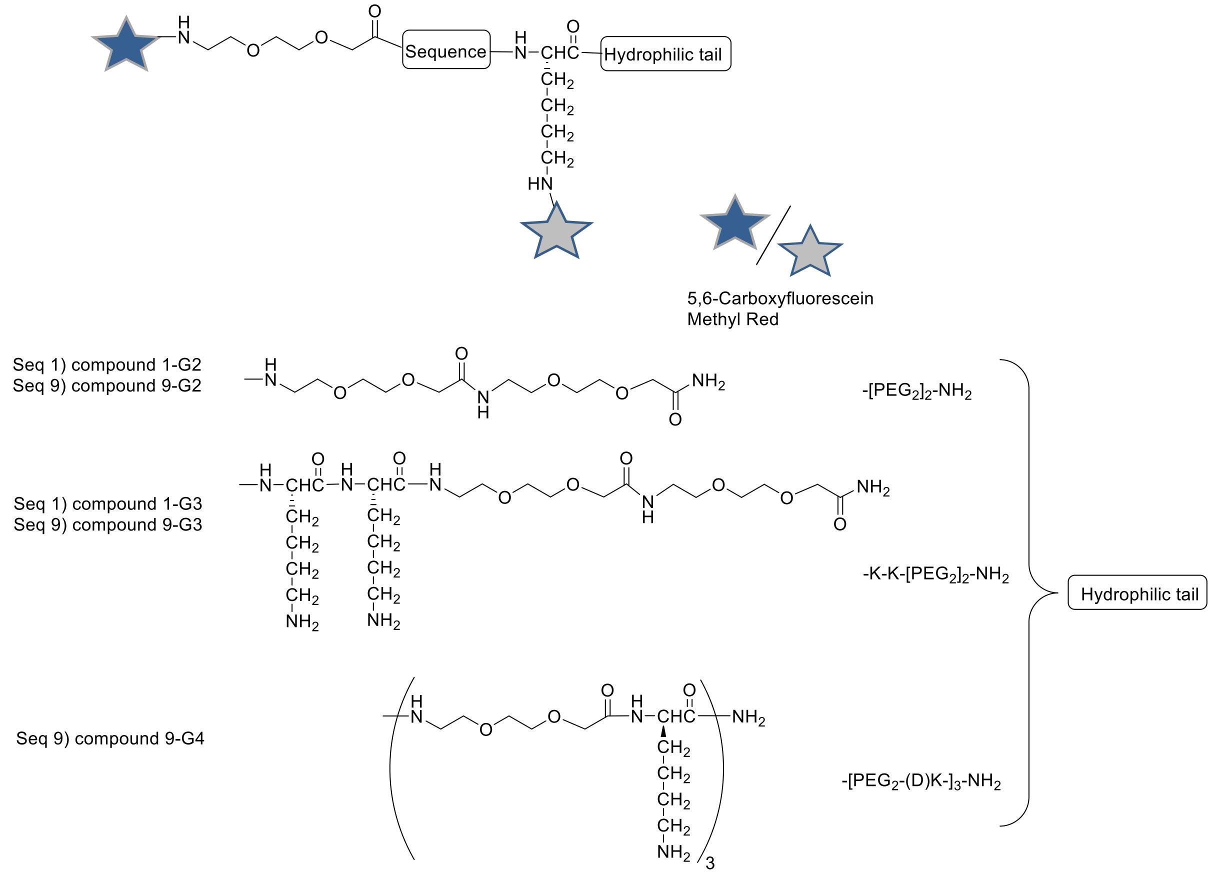

Figure S22. Structures of compounds in Generation-2, -3 and -4

<u>a) MMPs vs Plasmin selectivity:</u>

Experiments with MMP-9,-13 and plasmin were carried out in parallel with the selected sequences. Comparison of results provided by compounds 1-G2 and 9-G2 indicated that sequence 1 was cleaved by plasmin while sequence 9 was totally plasmin resistant. MALDI TOF MS analysis (Figure S23-S24) was carried out and plasmin cleavage site for sequence 1 was identified as (-G-P-K↑G-L-K-G-). Attempts to make a resistant version replacing the lysine residue with D-aminoacids (-G-P-(D)K-G-L-K-G-) resulted in the failure of enzymatic recognition by the MMPs.

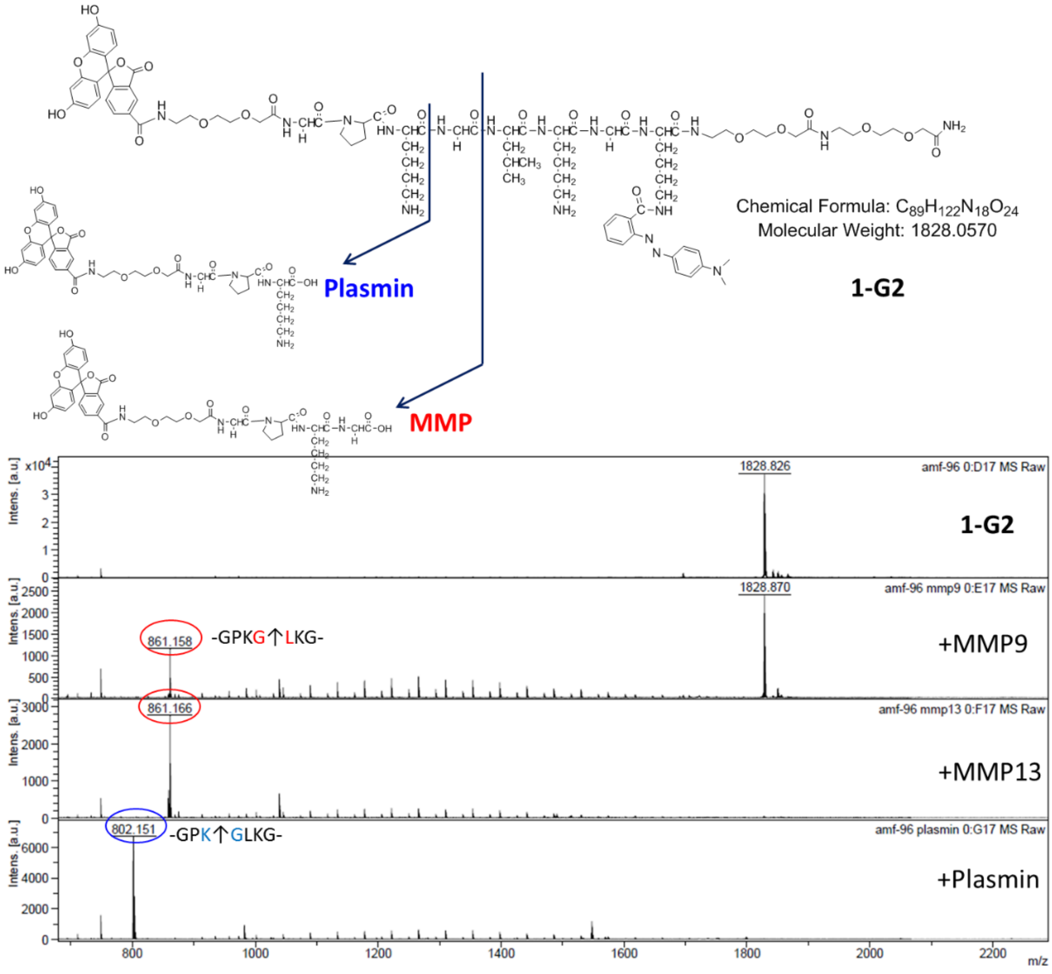

Figure S23. MALDI TOF MS spectra of compound 1-G2 with the different MMPs and Plasmin

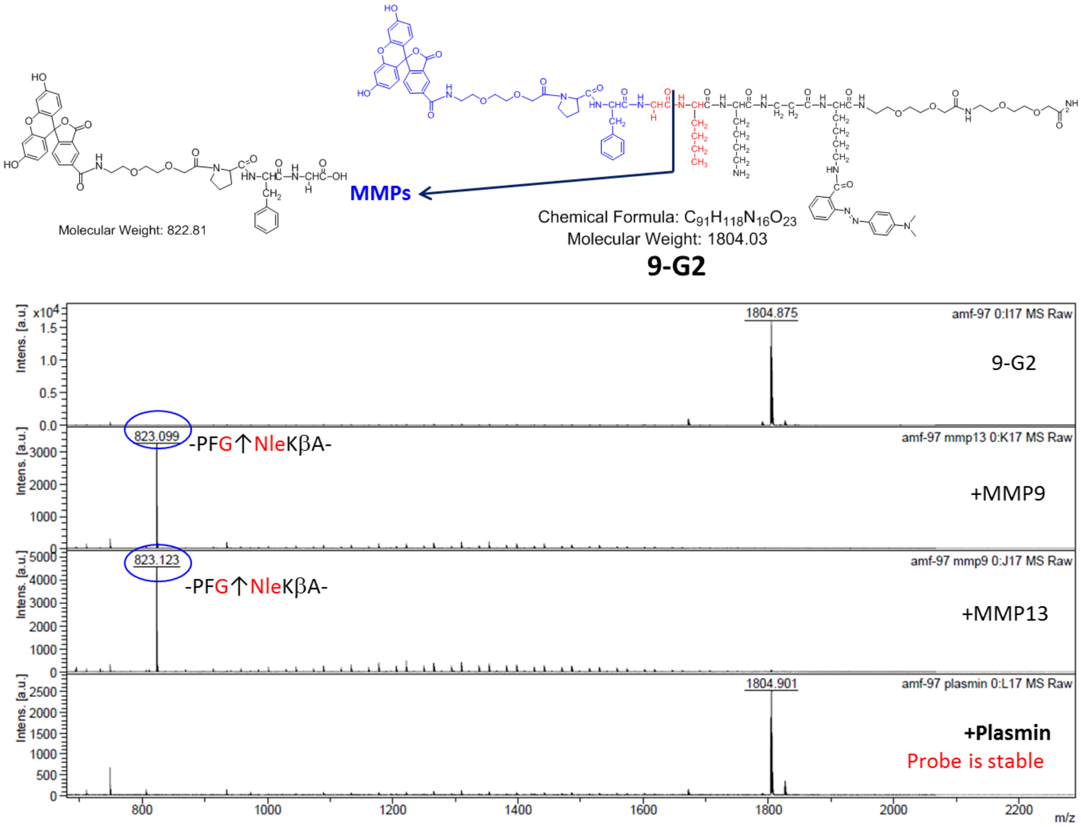

Figure S24. MALDI TOF MS spectra of compound 9-G2 with the different MMPs and Plasmin

b) Hydrophilic tail optimization:

The combination of PEG units with Lysine increased the aqueous solubility progressively with each generation although the fragment –K-K-$(PEG_2)_2$-NH_2 used in generation 3 (compounds 1-G3 and 9-G3) was unspecifically cleaved when the probes were assayed in tissue homogenate (Figure S25). A final iteration with the plasmin resistant sequence 9 (compound 9-G4) was synthesized containing as a hydrophilic tail alternate *D*-Lys as a non-natural aminoacid and PEG units resulting in good solubility and stability as confirmed by MALDI TOF MS and HPLC analysis. The fluorescence increase was selective for MMPs over plasmin (Fig S26).

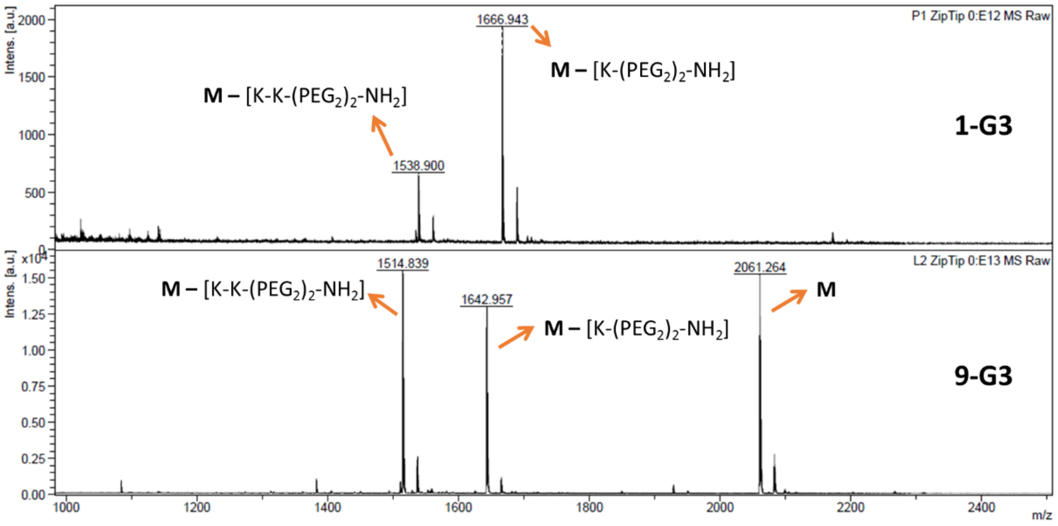

Figure S25. MALDI TOF MS spectra for 1-G3 (upper) and 9-G3 (lower) when incubated with healthy (homogenised) human lung tissue showing cleavage of the hydrophilic tail ↑–K↑-K-$(PEG_2)_2$-NH_2

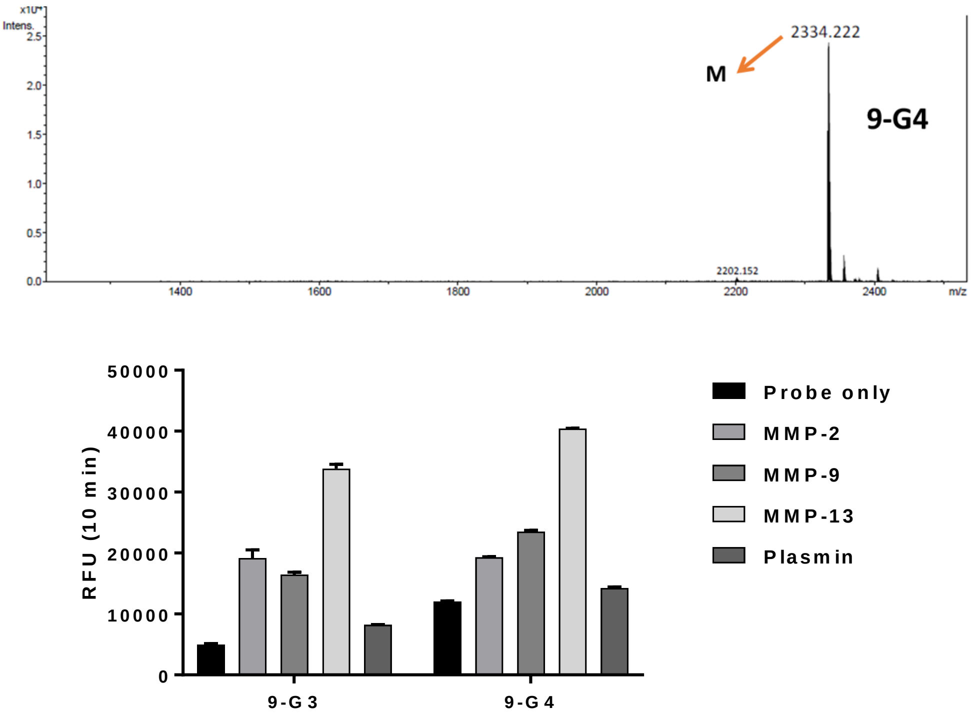

Figure S26. (upper) MALDI TOF MS spectra of 9-G4 when incubated with homogenised healthy human lung tissue showing stabilising effect of the hydrophilic tail –[PEG$_2$-(D)K]$_3$-NH$_2$. (lower) Fluorescence signal of compounds 9-G3 and 9-G4 in the presence of different enzymes.

In summary from these studies the optimized structure for the MMP probe was selected as PFGNleKβA attached to the hydrophilic tail -[PEG$_2$-(D)K]$_3$-NH$_2$ and this was used to construct the optimized dual-probe. The widley used MMP peptide GPKGLKG was shown to be non-viable due to its cleavage by endogenous enzymes such as Plasmin. Whilst the initial MMP-peptide sequence was chosen for gelatinases MMP-2 and MMP-9 selectivity[15] our final modified peptide sequence was also highly selective for MMP-13, a collagenase which along with MMP-2 and MMP-9 is upregulated within inflammatory microenvironments.[16] It is not wholly surprising that our peptide sequence was activated by these three different MMPs as they share common targets such as gelatin and several collagen sub-types.[17]

SYNTHESIS OF MMP PROBES

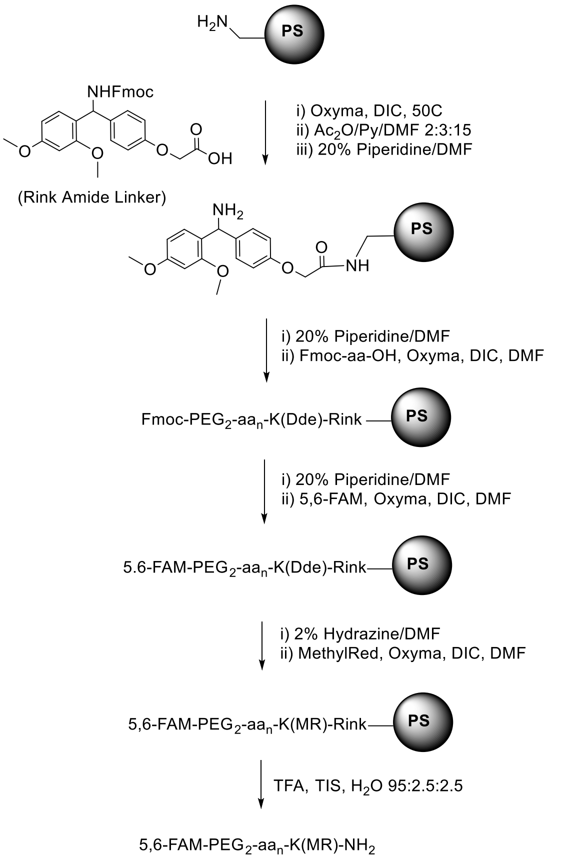

SEQ ID NO.	Sequence
1	**G-P-K-*G-L*-K-G**
2	G-P-K-G-(D)L-K-G
3	G-P-K-*G-I*-K-G
4	G-P-K-*G-Nle*-K-G
5	P-F-*G-M*-K-βA
6	P-F-G-(D)M-K-βA
7	P-F-*G-L*-K-βA
8	P-F-*G-I*-K-βA
9	**P-F-*G-Nle*-K-βA**
10	P-Cha-*G-M*-F-G
11	P-Cha-*G-M*-W-G
12	P-Cha-*G-M*-Y(Me)-G
13	P-Cha-*G-M*-Y-G
14	P-Cha-*G-M*-K-βA-G
15	P-Cha-*G-M*-H-G
16	P-Cha-*G-M*-K-G

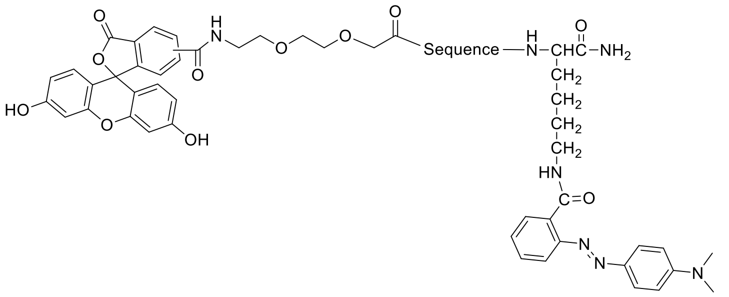

Scheme S6. MMP substrate optimization. Synthesis of the library of the Generation 1 FRET peptides was carried out according to the general methods described above.

Characterisation Table of MMP probes library (Generation 1 to Generation 4)

Probe	Structure	m/z_{calc} (Da) $[M+H]^+$	MALDI-TOF m/z (Da)	HPLC t_R (min)
**1-G1**	FAM-PEG$_2$-(Seq1)-K(MR)-NH$_2$	1537.74	1538.20	6.357[b]
**2-G1**	FAM-PEG$_2$-(Seq2)-K(MR)-NH$_2$	1537.74	1538.30	6.456[b]
**3-G1**	FAM-PEG$_2$-(Seq3)-K(MR)-NH$_2$	1538.71	1538.21	6.259[b]
**4-G1**	FAM-PEG$_2$-(Seq4)-K(MR)-NH$_2$	1538.71	1538.31	6.150[b]
**5-G1**	FAM-PEG$_2$-(Seq5)-K(MR)-NH$_2$	1530.66	1531.80	5.390[c]
**6-G1**	FAM-PEG$_2$-(Seq6)-K(MR)-NH$_2$	1532.76	1532.58	6.342[b]
**7-G1**	FAM-PEG$_2$-(Seq7)-K(MR)-NH$_2$	1514.72	1514.66	6.394[b]
**8-G1**	FAM-PEG$_2$-(Seq8)-K(MR)-NH$_2$	1514.72	1514.76	6.218[b]
**9-G1**	FAM-PEG$_2$-(Seq9)-K(MR)-NH$_2$	1514.72	1514.68	6.330[b]
**10-G1**	FAM-PEG$_2$-(Seq10)-K(MR)-NH$_2$	1542.65	1543.70	6.744[c]
**11-G1**	FAM-PEG$_2$-(Seq11)-K(MR)-NH$_2$	1582.79	1582.60	6.669[c]
**12-G1**	FAM-PEG$_2$-(Seq12)-K(MR)-NH$_2$	1573.70	1573.60	6.714[c]
**13-G1**	FAM-PEG$_2$-(Seq13)-K(MR)-NH$_2$	1558.65	1558.60	6.307[c]
**14-G1**	FAM-PEG$_2$-(Seq14)-K(MR)-NH$_2$	1537.70	1538.70	5.552[c]
**15-G1**	FAM-PEG$_2$-(Seq15)-K(MR)-NH$_2$	1532.65	1532.60	5.522[c]
**16-G1**	FAM-PEG$_2$-(Seq16)-K(MR)-NH$_2$	1524.69	1524.70	5.586[c]
**1-G2**	FAM-PEG$_2$-(Seq1)-K(MR)-PEG$_2$-PEG$_2$-NH$_2$	1829.06	1829.13	4.674[c]
**9-G2**	FAM-PEG$_2$-(Seq9)-K(MR)-PEG$_2$-PEG$_2$-NH$_2$	1827.02[a]	1827.09[a]	4.773[c]
**1-G3**	FAM-PEG$_2$-(Seq1)-K(MR)-K-K-[PEG$_2$]$_2$-NH$_2$	2085.41	2085.31	3.254[c]
**9-G3**	FAM-PEG$_2$-(Seq9)-K(MR)-K-K-[PEG$_2$]$_2$-NH$_2$	2061.39	2061.39	3.958[c]
**9-G4**	MR-PEG$_2$-(Seq9)-K(FAM)-[PEG$_2$-k]$_3$-NH$_2$	2334.72	2334.22	3.823[c]

Table S5. [a] $[M+Na]^+$; Analytical HPLC: Flow rate of 1 mL/min and detection at 254, 495 nm and by evaporative light scattering. [b] Elution with $H_2O/CH_3CN/HCOOH$ (95/5/0.1) to $H_2O/CH_3CN/HCOOH$ (5/95/0.1), over 10 min, holding at 95% ACN for 4 min; [c] Elution with $H_2O/CH_3CN/HCOOH$ (95/5/0.1) to $H_2O/CH_3CN/HCOOH$ (5/95/0.1), over 6 min, holding at 95% ACN for 2 min.

REFERENCES

[1] B. Amulic, C. Cazalet, G. L. Hayes, K. D. Metzler, A. Zychlinsky, *Annual Review of Immunology* **2012**, *30*, 459-489.

[2] a) J. Pugin, M. C. Widmer, S. Kossodo, C. M. Liang, H. L. n. Preas, A. F. Suffredini, *Am J Respir Cell Mol Biol* **1999**, *20*, 458-464; b) V. C. Ardi, T. A. Kupriyanova, E. I. Deryugina, J. P. Quigley, *Proc Natl Acad Sci U S A* **2007**, *104*, 20262-20267.

[3] G. Ferry, M. Lonchampt, L. Pennel, G. de Nanteuil, E. Canet, G. C. Tucker, *FEBS Lett* **1997**, *402*, 111-115.

[4] G. T. Gerotziafas, I. Elalamy, F. Depasse, E. Perzborn, M. M. Samama, *J Thromb Haemost* **2007**, *5*, 886-888.

[5] S. Takahashi, W. Piao, Y. Matsumura, T. Komatsu, T. Ueno, T. Terai, T. Kamachi, M. Kohno, T. Nagano, K. Hanaoka, *Journal of the American Chemical Society* **2012**, *134*, 19588-19591.

[6] M. Gude, J. Ryf, P. D. White, *Letters in Peptide Science* **2002**, *9*, 203-206.

[7] J. J. Diaz-Mochon, L. Bialy, M. Bradley, *Org. Lett.* **2004**, *6*, 1127-1129.

[8] D. P. Nguyen, H. Lusic, H. Neumann, P. B. Kapadnis, A. Deiters, J. W. Chin, *Journal of the American Chemical Society* **2009**, *131*, 8720-8721.

[9] R. C. Brown, Z. Li, A. J. Rutter, X. Mu, O. H. Weeks, K. Smith, I. Weeks, *Organic & Biomolecular Chemistry* **2009**, *7*, 386-394.

[10] M. Lopalco, E. N. Koini, J. K. Cho, M. Bradley, *Org. Biomol. Chem.* **2009**, *7*, 856-859.

[11] V. Hong, S. I. Presolski, C. Ma, M. G. Finn, *Angewandte Chemie International Edition* **2009**, *48*, 9879-9883.

[12] A. G. Rossi, J. C. McCutcheon, N. Roy, E. R. Chilvers, C. Haslett, I. Dransfield, *The Journal of Immunology* **1998**, *160*, 3562-3568.

[13] N. Krstajić, B. Mills, I. Murray, A. Marshall, D. Norberg, T. H. Craven, P. Emanuel, T. R. Choudhary, G. O. S. Williams, E. Scholefield, A. R. Akram, A. Davie, N. Hirani, A. Bruce, A. Moore, M. Bradley, K. Dhaliwal, *J. Biomed. Opt* **2018**, *23*, 076005.

[14] K. Dhaliwal, E. Scholefield, D. Ferenbach, M. Gibbons, R. Duffin, D. A. Dorward, A. C. Morris, D. Humphries, A. MacKinnon, T. S. Wilkinson, W. A. H. Wallace, N. van Rooijen, M. Mack, A. G. Rossi, D. J. Davidson, N. Hirani, J. Hughes, C. Haslett, A. J. Simpson, *American Journal of Respiratory and Critical Care Medicine* **2012**, *186*, 514-524.

[15] A. Prudova, U. auf dem Keller, G. S. Butler, C. M. Overall, *Molecular & Cellular Proteomics* **2010**, *9*, 894-911.

[16] a) G. Shay, C. C. Lynch, B. Fingleton, *Matrix Biology* **2015**, *44–46*, 200-206; b) E. Hadler-Olsen, J. O. Winberg, L. Uhlin-Hansen, *Tumour Biol* **2013**, *34*, 2041-2051; c) L. Nissinen, V.-M. Kähäri, *Biochimica et Biophysica Acta (BBA) - General Subjects* **2014**, *1840*, 2571-2580.

[17] C. M. Overall, *Mol Biotechnol* **2002**, *22*, 51-86.
